# Successful and sustained implementation of a behaviour-change informed strategy for emergency nurses: a multicentre implementation evaluation

**DOI:** 10.1186/s13012-024-01383-7

**Published:** 2024-07-29

**Authors:** Kate Curtis, Belinda Kennedy, Julie Considine, Margaret Murphy, Mary K. Lam, Christina Aggar, Margaret Fry, Ramon Z. Shaban, Sarah Kourouche

**Affiliations:** 1 Susan Wakil School of Nursing and Midwifery, Faculty of Medicine and Health, RC Mills Building, University of Sydney, Camperdown, NSW 2006 Australia; 2 Emergency Services, Illawarra Shoalhaven Local Health District, Wollongong Hospital, Crown St, Wollongong, NSW Australia; 3 School of Nursing and Midwifery and Centre for Quality and Patient Safety Experience in the Institute for Health Transformation, Deakin University, Geelong, VIC Australia; 4 Centre for Quality and Patient Safety Research – Eastern Health Partnership, Box Hill, VIC Australia; 5 Western Sydney Local Health District, North Parramatta, NSW 2141 Australia; 6 School of Health and Biomedical Sciences, RMIT University, Melbourne, VIC Australia; 7 Northern NSW Local Health District, Southern Cross University, Lismore, Australia; 8 Sydney Faculty of Health, University of Technology, Ultimo, NSW Australia; 9 Northern Sydney Local Health District, St Leonards, NSW Australia; 10 Sydney Infectious Diseases Institute, Faculty of Medicine and Health, The University of Sydney, Camperdown, NSW 2006 Australia; 11 Research and Education Network, Western Sydney Local Health District, Westmead, NSW 2145 Australia; 12 New South Wales Biocontainment Centre, Western Sydney Local Health District, Westmead, NSW 2145 Australia

**Keywords:** Emergency nursing, Emergency department, Patient assessment, Randomized control trial, Clinical deterioration, Implementation, Education, Behaviour change, Patient safety, Evidence-Based Nursing, Emergency Nursing, Implementation Science, Emergency Service, Hospital, Patient Safety, Program Evaluation, Randomized Controlled Trial [Publication Type], Clinical Deterioration, Education, Nursing

## Abstract

**Background:**

Implementing evidence that changes practice in emergency departments (EDs) is notoriously difficult due to well-established barriers including high levels of uncertainty arising from undifferentiated nature of ED patients, resource shortages, workload unpredictability, high staff turnover, and a constantly changing environment. We developed and implemented a behaviour-change informed strategy to mitigate these barriers for a clinical trial to implement the evidence-based emergency nursing framework HIRAID^®^ (History including Infection risk, Red flags, Assessment, Interventions, Diagnostics, communication, and reassessment) to reduce clinical variation, and increase safety and quality of emergency nursing care.

**Aim:**

To evaluate the behaviour-change-informed HIRAID^®^ implementation strategy on reach, effectiveness, adoption, quality (dose, fidelity) and maintenance (sustainability).

**Methods:**

An effectiveness-implementation hybrid design including a step–wedge cluster randomised control trial (SW-cRCT) was used to implement HIRAID^®^ with 1300 + emergency nurses across 29 Australian rural, regional, and metropolitan EDs. Evaluation of our behaviour-change informed strategy was informed by the RE-AIM Scoring Instrument and measured using data from (i) a post HIRAID^®^ implementation emergency nurse survey, (ii) HIRAID^®^ Instructor surveys, and (iii) twelve-week and 6-month documentation audits. Quantitative data were analysed using descriptive statistics to determine the level of each component of RE-AIM achieved. Qualitative data were analysed using content analysis and used to understand the ‘how’ and ‘why’ of quantitative results.

**Results:**

HIRAID^®^ was implemented in all 29 EDs, with 145 nurses undertaking instructor training and 1123 (82%) completing all four components of provider training at 12 weeks post-implementation. Modifications to the behaviour-change informed strategy were minimal. The strategy was largely used as intended with 100% dose and very high fidelity. We achieved extremely high individual sustainability (95% use of HIRAID^®^ documentation templates) at 6 months and 100% setting sustainability at 3 years.

**Conclusion:**

The behaviour-change informed strategy for the emergency nursing framework HIRAID^®^ in rural, regional, and metropolitan Australia was highly successful with extremely high reach and adoption, dose, fidelity, individual and setting sustainability across substantially variable clinical contexts.

**Trial registration:**

ANZCTR, ACTRN12621001456842. Registered 25 October 2021.

**Supplementary Information:**

The online version contains supplementary material available at 10.1186/s13012-024-01383-7.

Contributions to the literature

- Evaluating methods to translate evidence effectively and rapidly to emergency practice is crucial to keep pace with increasing demand for emergency care. Strategies to overcome implementation barriers improve clinical trial performance and intervention implementation in the complex emergency setting.
- Our behaviour-change strategy enabled maximum, sustained uptake of an intervention in a variety of emergency settings despite the COVID-19 pandemic and catastrophic flooding.
- The Mechanisms of Action “Optimism” and “Social Professional Role and Identity” addressed barriers related to feelings of a lack of support by management and a lack of time to change practice, previously reported as having no definite links.

## Background

**Fig. 1:**
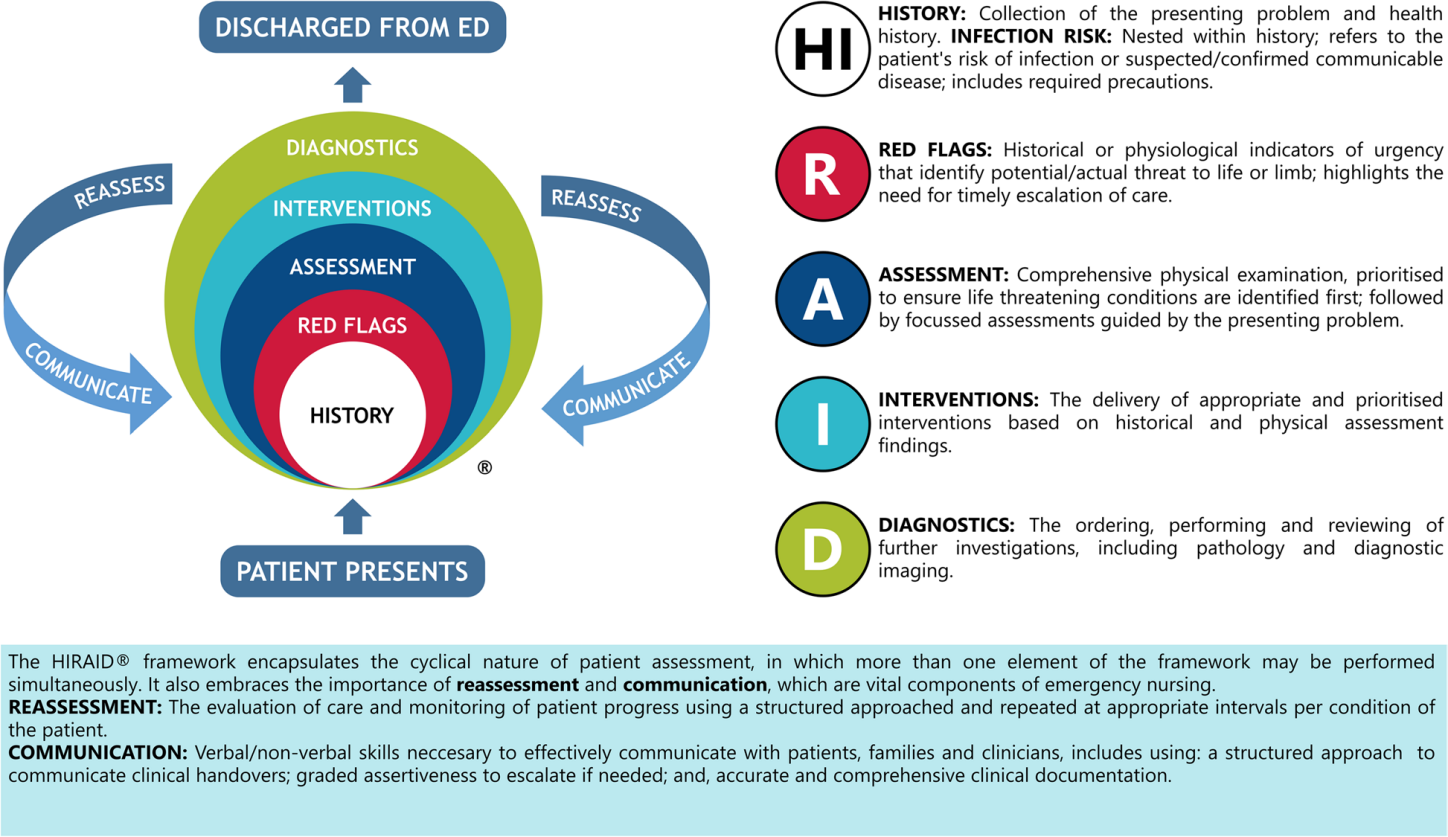
HIRAID^®^ Emergency Nursing Framework [2]

As demand for emergency care increases globally so too does the need for timely recognition and treatment of the acutely ill and injured for high quality and safe healthcare [1]. Emergency nurses are the first emergency department (ED) clinicians to assess and manage patients, and their practice is fundamental to patient safety. Emergency nurses are responsible for the initial and ongoing assessment, management, and safety of patients of all ages, with varying degrees of clinical urgency and severity. To enable safe and quality emergency nursing care, HIRAID^®^ was developed [History including Infection risk, Red flags, Assessment, Interventions, Diagnostics, communication, and reassessment)] [2–4] (Fig. 1). HIRAID^®^ is an emergency nursing framework used by emergency nurses for any patient presentation and the only validated framework designed to enable emergency nurses to systematically assess and manage ED patients [5]. In feasibility, efficacy and replicability studies, use of HIRAID^®^ reduced clinical deterioration related to emergency nursing care, improved clinical documentation [6], handover [3] and reduced treatment costs [7].

Successful and sustained implementation of any intervention in health care is challenging [8]. Many large trials have failed to effectively change emergency nursing practice [9, 10]. EDs have well-established barriers of high levels of uncertainty arising from the undifferentiated nature of patients, chronic resource shortages and limitations, workload unpredictability, high staff turnover and constantly changing environments. They have unique external (policies, resources, and incentives) and internal (local organizational readiness, infrastructures, and workflows) [11] contextual characteristics, including micropolitics [12], that influence implementation. Early planning including the identification of particular ED characteristics and associated factors that influence clinician behaviour is necessary for the successful implementation of an intervention [13]. Strategies to achieve successful clinician behaviour change, clinical trial performance, and intervention implementation in complex healthcare settings such as EDs can be successful with behavioural optimisation [14–17]. Put simply, evaluating what works in the emergency context is critical to future implementation.

To implement HIRAID^®^ in 29 Australian rural, regional and metropolitan EDs as part of an effectiveness-implementation hybrid study [18], we developed and executed a behaviour-change implementation strategy to mitigate the above barriers using a systematic process guided by the behaviour change wheel and theoretical domains framework [19]. Developed with end users, implementation experts and stakeholders a multifaceted implementation strategy was developed with 21 behaviour change techniques (BCT) from the behaviour change technique taxonomy (BCTT) [20]. Strategies included an educational program—HIRAID^®^ Instructor (train-the-trainer) and Provider courses including eLearning module, and face-to-face education; visual prompts in the electronic medical records such as a template to document the HIRAID^®^ assessment; local change champions to support, model in clinical practice; a video featuring clinical leaders and individualised audit and feedback. More detail is provided with the results and elsewhere (Curtis K, Kennedy B, Considine J, Murphy M, Kourouche S, Lam MK, Shaban RZ, Aggar C, Hughes JA, Fry M: Design of a data-driven implementation strategy to optimise clinician behaviour change at scale in complex clinical environments: A multicentre emergency care study, under review).

Prior to analyses to measure the effectiveness of the intervention on patient and health services outcomes, the effectiveness of the implementation strategy required evaluation, particularly around implementation fidelity, which is the extent to which an intervention is delivered per the intended implementation plan [21, 22]. Fidelity is critical to the internal and external validity of implementation [22]. Implementation science theories and models such as the RE-AIM framework (Reach, Effectiveness, Adoption, Implementation (dose/fidelity) and Maintenance / Sustainability) provide a robust framework for evaluating implementation outcomes [23]. Improved implementation outcomes can directly contribute to better patient outcomes [24]. The aim of this study was to evaluate the behaviour-change informed HIRAID^®^ implementation strategy on reach, effectiveness, adoption, quality (dose, fidelity) and maintenance (sustainability).

## Methods

We used a type II effectiveness-implementation hybrid design [25] including a step–wedge cluster randomised control trial (SW-cRCT) to evaluate: i) the patient outcomes of HIRAID^®^ implementation at scale (effectiveness) and ii) strategies that enable optimal HIRAID^®^ uptake (implementation). The hybrid approach allows testing of the implementation strategy at the same time as observing the outcomes of the intervention [26]. The purpose of this paper is evaluation of the implementation strategy, the patient outcomes and cost effectiveness will be reported separately to allow for a more comprehensive report. The HIRAID^®^ implementation strategy evaluation plan was developed during study design guided by the RE-AIM framework [27] and Scoring Instrument [28] (Table 1).

**Table 1:** RE-AIM evaluation plan to evaluate HIRAID^®^ implementation strategy

RE-AIM domain and definition	Intended outcome	Measure / Indicator	Data Source
**Reach** The number, proportion, and representativeness of individuals willing to participate in the HIRAID^®^ program [29]	Individual: > 80% of nurses from all 29 EDs complete HIRAID^®^ training program representing novice to experienced nurses from all ED types	% of eligible nurses that completed (i) any training (ii) all training (iii) % sites represented % of eligible nurses that (iv) completed post intervention survey (v) % sites represented (vi) range of years of experience of those attending	Study records Staff survey
	Setting: 100% sites have a senior nurse “Instructor” complete HIRAID^®^ Instructor program	Proportion of eligible sites that had a nurse complete instructor training	Study records
Effectiveness* Effectiveness assesses whether the targeted behavioural outcome was achieved and changes to quality of life (QOL) or other important outcomes [1] *Patient, health service and costing effectiveness to be reported elsewhere. Purpose of this paper is to evaluate implementation strategy on implementation	> 80% nurse participant opinion on the usefulness of HIRAID^®^ Sub-group analyses to draw conclusions about how different subpopulations responded to the intervention Percent attrition Results for at least one follow-up	% eligible nurses who indicated HIRAID^®^ helps: (i) remember all parts of a full assessment (ii) teach new emergency nurses how to assess and manage patients	Staff survey + free text comments
	Uptake of BCTTs – opinion from staff on particular Behaviour change techniques (BCTs)		Survey Free text
**Adoption** (settings level) The number of sites eligible and willing to initiate a program, and why (Individual level) Delivery staff invited to participate, and characteristics of participants) and intervention adoption rate	100% of eligible sites implementing HIRAID^®^ Compare their characteristics to nonparticipating settings (N/A)	% of eligible EDs that implement HIRAID^®^	Study records
	100% Senior nurse “Instructor” from each site complete HIRAID^®^ Instructor program	% of sites that have staff attend instructor course	Study records
	> 80% use of HIRAID^®^ templates to document patient assessment	% patients who had HIRAID^®^ documentation when needed	Audits uptake 6 weeks, 12 weeks Staff survey Free text comments explaining why or why not used
**Implementation quality (dose and fidelity)** Consistency of implementation across settings and within planned time frames to the intervention strategy, including time and cost as intended	Dose: > 90% of 21 BCTTs used [30] > 80% within timeframe	Proportion of BCTTs implemented by total number planned - Number of instructor and provider courses delivered at how many sites to how many nurses - eLearning % completions - Face to face sessions % completions - Number face to face sessions - Number champions at each site - Time frame delivered per protocol	Study records
	Fidelity: > 80% of perfect delivery by the HIRAID^®^ team at (i) Instructor course (setting) (ii) Provider course (individual). Did each Instructor deliver the same material? < 30% adaptations made to BCTs and implementation strategies and reasons	% of elements delivered in Instructor program and materials delivered % of 15 prescribed elements delivered all the time, most of the time, hardly ever % adaptations made to HIRAID^®^ delivery or implementation strategy (quant and qual) % of elements used (doc templates, memo, flip card etc.)	Instructor course delivery Study 15 item Instructor survey (quant) Implementation logs (qual) HIRAID^®^ Instructors completed an intervention fidelity scoring sheet that asks to what degree the behaviour change mechanisms were implemented [22]
	Quality: Level of staff satisfaction with the delivery of education related to the HIRAID^®^ framework	The proportion of staff surveyed that rated the education to be delivered in an engaging manner	Staff survey
	Cost of Intervention Measures of cost of implementation	Cost of face-to-face attendance by staff. Any additional resources needed or done within budget	Research partner letters
**Maintenance / Sustainability** The maintenance dimension assesses both individual-level behaviour change and organizational-level intervention sustainability [30]	> 90% sites have HIRAID^®^ embedded business as usual ≥ 6 month post study (Alignment with organizational mission) > 80% participants state they are using ≥ 6 months post intervention Qualitative measure of individual-level maintenance	Proportion of sites with HIRAID^®^ education program embedded in new nurse orientation program and how (which elements retained) Proportion of sites with HIRAID^®^ documentation templates in use Proportion of nurses saying they are using it 6 months post implementation	Instructor survey Local audit 2yrs post implementation Nursing survey

*Reach* was measured by the proportion of eligible nurses participating in the program, *effectiveness* through the end user (nurses) opinions on the usefulness of HIRAID^®^, and *adoption* by the proportion of eligible sites implementing HIRAID^®^. *Dose* was the consistency of exposure to HIRAID^®^ across settings and *fidelity,* the degree to which individual components and the overarching implementation strategy was delivered. *Sustainability* was determined through demonstration of ongoing HIRAID^®^ training program use post implementation, along with the proportion of nurses reporting they use HIRAID^®^ at ≥ 6 months (Table 1).

We used multiple methods to achieve this as recommended by Glasgow et al. [27]. Also guided by suggestions from the behaviour change wheel the BCT taxonomies was used to guide the evaluation plans for implementation fidelity. Quantitative data were collected to measure proportions and determine the level of each component of RE-AIM achieved. Qualitative data were used to understand the “how” and “why” of quantitative results, and any deviations or adaptations to the proposed implementation strategy.

Human Research Ethics approval was obtained for NSW sites from the Greater Western Human Research Ethics Committee (2020/ETH02164) and for Victorian sites through the Royal Brisbane & Woman’s Hospital Human Research Ethics Committee (2021/QRBW/80026). The study was prospectively registered with Australian New Zealand Clinical Trials Registry (ANZCTR) (Number ACTRN12621001456842). The study was conducted in accordance with the HREC approvals and the requirements of the 2018 National Health and Medical Research Council Australian Code for the Responsible Conduct of Research [31] and reported per Standards for Reporting Implementation Studies (StaRI) Statement [32] and CONSORT guidelines for reporting SW-RCTs [33].

### Implementation and settings

The HIRAID^®^ trial was a SW-cRCT across 29 EDs from two Australian States with over 1300 emergency nurses. The 29 EDs were grouped into four clusters per their local health district grouping (Table 2). Each cluster was randomised, commenced the trial in the control condition and sequentially crossed over to the intervention condition: the first cluster commenced November 9th, 2020. The “go live” comprised commencing HIRAID^®^ training for emergency nurses, availability of the HIRAID^®^ documentation templates, and formal instruction from hospital executives that HIRAID^®^ was starting to be used. The clusters represent a range of geographically and clinically diverse settings, from large teaching hospitals to some rural services without medical services onsite, with nursing staff required to call them in as required and/or use telehealth. The implementation strategy and development are described in detail elsewhere (Curtis K, Kennedy B, Considine J, Murphy M, Kourouche S, Lam MK, Shaban RZ, Aggar C, Hughes JA, Fry M: Design of a data-driven implementation strategy to optimise clinician behaviour change at scale in complex clinical environments: A multicentre emergency care study, under review) (Supplementary file 1).

**Table 2:** Trial Cluster, ED patient presentations, admissions, nursing staff and trial start date

Site	Context	ED patients per year	Admits via ED per year	Nurses	Description of LHD	Commencement date
Cluster1	10 rural, regional EDs	116,836	17,065	188	Spans 44,534km^2^ 200,000 + residents	09/11/2020
Cluster2	12 rural, regional EDs	213,307	40,539	385	Spans 20,732km^2^ 350,000 + residents	06/10/2021
Cluster3	4 metro EDs	202,516	67,975	394	Spans 780km^2^ 946,000 + residents	08/08/2022
Cluster4	3 metro EDs	169,465	47,320	410	Spans 2816km^2^ 750,000 + residents	10/11/2022
**Total**	**29 EDs**	**702,124**	**172,899**	**1,377**	**Range of remote, rural, regional and metro EDs**	

*Abbreviations*: *ED* Emergency departments, *km*^*2*^ Kilometre, *LHD* Local health district

### Participants

There were two groups of participants in this study: permanently employed emergency nurses (HIRAID^®^ providers), and HIRAID^®^ Instructors. All permanently employed emergency nurses were eligible and encouraged to undertake HIRAID^®^ Provider training to adopt HIRAID^®^ in their clinical practice. HIRAID^®^ Instructors were appointed at each site to aid in the consistent application and fidelity, most of whom were part of the existing nursing workforce at the cluster and in a position to support the change as part of their roles, such as the cluster Clinical Nurse Educators (CNEs) and Clinical Nurse Consultants (CNCs). Several of the smaller sites in NSW did not have a CNE, so we recruited a HIRAID^®^ Instructor in consultation with site managers. Site HIRAID^®^ Instructors were invited to attend weekly implementation team meetings and were asked about any implementation complications or adjustments.

### Data sources and collection procedures

There were three primary data sources: (i) a six-month post HIRAID^®^ implementation emergency nurse survey; (ii) HIRAID^®^ Instructor surveys; and (iii) six- and twelve-week documentation audits. Data from each source were mapped and integrated to RE-AIM outcomes per Table 2. Information from the project timeline as registered on the clinical trials website (ACTRN12621001456842) was also used [34].

#### Data source 1: Emergency nurse survey

An electronic cross-sectional survey of all permanently employed emergency nurses was conducted to ascertain their perspectives on the usability and implementation of HIRAID^®^ six months post implementation “go live” at each ED. Casual staff were not approached as they were generally not included in the training program and a transient workforce. The first section of the survey collected participant characteristics (place of work and years of nursing experience). This was followed by 10 multiple-choice questions to ascertain their perspective on strategies used to introduce HIRAID^®^ to their ED. For example, “Do you use the HIRAID^®^ documentation templates in ED for your initial documentation (post triage)? If not, why not?”. The questions in the survey were used to identify if staff used the implementation strategies (reach), how often they were used (dose), and about how the strategies were delivered (adaptations) and were adapted from a previous survey [35]. All questions had free text options for participants to explain their response (Supplementary material 2).

Survey data were collected and managed using REDCap™ (Research Electronic Data Capture) a secure, web-based software platform and electronic data capture tool hosted at the University of Sydney [36]. The survey was tested for face validity during a pilot phase and minor changes to formatting and flow were made with testing by the research team and key stakeholders at the sites. Surveys, with a participant information and consent form clearly stating the voluntary nature of the survey and anonymity of survey responses, were distributed to all eligible staff at each cluster by departmental managers and kept open for four weeks. Reminders were sent weekly. An incentive was offered for survey completion (a variety of $20 AUD vouchers suitable for participant’s location, for example a local cafe) as a key strategy known to increase response rates [37].

#### Data source 2: HIRAID^®^ Instructor surveys

There were two HIRAID^®^ Instructor surveys to identify the dose and fidelity of the implementation strategies. Firstly, a five-question survey was distributed at the completion of the Instructor course to determine if participants felt they had a good understanding of what was expected of them as a HIRAID^®^ Instructor and that they were confident in their ability to deliver HIRAID^®^ training using a 5-point Likert scale (Often/ Always, Sometimes, Rarely, Never, not sure). A second electronic survey was distributed at 12 weeks post-implementation asking to what degree the 21 behaviour change techniques were implemented [22] (not used at all, rarely used, sometimes used and used most or all the time). Adaptations to the way the education program was delivered was also scored 1 (nil changes) to 3 (major changes from plan) (Supplementary material 1) Understanding the adaptations was important in evaluating the strategies relevant to the contexts.

Following testing for face validity, these survey data were also managed using REDCap™ [36]. Reminders were sent weekly for 4 weeks. An incentive was offered to participants on survey completion (30AUD supermarket voucher) [37].

#### Data source 3: Documentation audits

At six, 12 and 24 weeks post “go live” at each site, a documentation audit was conducted by the onsite research nurses to determine if nurses were using HIRAID^®^ documentation templates to record their initial patient assessment. Three consecutive initial nursing patient assessments in the medical record, from every nurse rostered on any shift during a seven-day period, were reviewed at all sites to determine if nurses used the HIRAID^®^ template to record their initial nursing assessment, and, that the correct template was used (for example, paediatric template for a paediatric patient). The reports were distributed to the lead investigators at each site for feedback.

### Data analysis

Quantitative data were extracted and imported into Statistics Package for Social Sciences (SPSS) Version 22.0 [38] for analyses. Numeric data was analysed descriptively using median and interquartile range (IQR) due to the skewed distribution, while categorical data was analysed descriptively using counts and percentages. Kruskal–Wallis test was used to examine the overall relationship between experience and site, with Mann–Whitney test was used for pairwise comparisons. Chi-square test was used to examine the association between position and qualification with site. Overall significance set at 0.05. Bonferroni adjustment applied to multiple comparisons. Pairwise comparisons was significant at 0.008.

Qualitative data from surveys, were imported into NVivo™ v14 [39] and analysed collectively using the Graneheim [40] method of inductive content analysis by one author (KC). The text for each of the questions was condensed and coded into meaning units then grouped codes with similarities together into sub-themes [41]. Abstraction and interpretation of the latent content was kept low as the content was brief. Broader themes were grouped from the sub-themes considering the meanings. Findings from the quantitative and qualitative components were merged in a table to simultaneously array the quantitative and quantitative results using a convergent mixed methods approach [42]

Implementation fidelity was assessed by examining percentage of agreement calculated from the HIRAID^®^ Instructor survey that captured the degree to which each implementation strategy and Behaviour change techniques (BCTs) were delivered as intended [20]. Although there is no actual agreement to what comprises an acceptable percentage of agreement [43], high fidelity may be represented by a percentage of agreement of 80–100% and low integrity 50% or less [44]. We selected a goal of 80% or more of Instructors indicating that the intervention was delivered as intended (i.e. a score of 4) to indicate high fidelity, 60–80% moderate delivery, and 59% or less for low fidelity [45]. Percentage of agreement was calculated, where possible to evaluate setting and individual results. Subgroup comparisons between clusters were conducted.

## Results

All 29 sites agreed to participate and adopt HIRAID^®^, held on site training, and allocated non clinical time for their 1377 nurses to complete HIRAID^®^ Provider training. Emergency nurses (39% *n* = 534) completed the six-month post-implementation survey and had a median(IQR) 4(1–11) years ED experience. Respondents from the two rural regional clusters were more experienced with a median(IQR) 5 (2–13) and 8 (2–15) years emergency nursing) compared to the metropolitan clusters who had a median(IQR) of 2 (1–5) and 3 (1–10) years emergency nursing experience (KW(df3) = 49.7; *p* < 0.0001). More rural regional respondents had post graduate nursing qualifications (63.9% and 71.3% vs 43% and 58.1%, (X2(df9) = 27.7, *p* = 0.001). Over half the designated Instructors [64% (*n* = 90/145)] completed the Instructor survey, representing all clusters, although this proportion is likely closer to 70% as several instructors left the ED or changed roles during the implementation as noted in the implementation log managed by the lead investigator for each cluster (Table 3).

**Table 3:** Post HIRAID^®^ implementation nursing survey respondent characteristics overall and by cluster

Question	Overall (*N* = 534)	Cluster 1 (*N* = 97)	Cluster 2 (*N* = 122)	Cluster 3 (*N* = 160)	Cluster 4 (*N* = 155)	Significance
**Position**						0.062
Registered Nurse	420 (78.7%)	70 (72.2%)	93 (76.2%)	138 (86.3%)	119 (76.8%)	
Leadership role	93 (17.4%)	24 (24.7%)	21 (17.2%)	19 (11.9%)	29 (18.7%)	
Other – NP / EEN	21 (3.9%)	3 (3.1%)	8 (6.6%)	3 (1.9%)	7 (4.5%)	
**Experience (median IQR)**						
Years nursing	7 (3–15)	10 (5–22)	12 (6–22)	4 (2–7)	6 (3–13)	< 0.0001
Year in ED	4 (1–11)	5 (2–13)	8 (2–15)	2 (1–5)	3 (1–10)	< 0.0001
**Highest post grad qual**						0.001
None	226 (42.3%)	35 (36.1%)	35 (28.7%)	91 (56.9%)	65 (41.9%)	
Grad Certificate	214 (40.1%)	42 (43.3%)	58 (47.5%)	54 (33.8%)	60 (38.7%)	
Grad Diploma	59 (11%)	13 (13.4%)	18 (14.8%)	10 (6.3%)	18 (11.6%)	
Masters	35 (6.6%)	7 (7.2%)	11 (9%)	5 (3.1%)	12 (7.7%)	

### Reach

The setting and individual reach of the intervention was high. At least one senior nurse from each site completed Instructor program equating to 100% setting reach. Individual reach was also high. At the end of the 12 week implementation period, 91% (*n* = 1255) of eligible nurses had completed at least one aspect of the HIRAID^®^ training program. The majority of nurses [82% (*n* = 1123)], representing all 29 sites had completed the full HIRAID^®^ training program. This increased to 96% (*n* = 1323) at 6 months. A larger proportion of eligible nurses from the metropolitan clusters completed the training compared to those from the rural / regional clusters. Data were not collected on the characteristics of participants to enable comparison with nonparticipants (Table 4).

**Table 4:** Setting and individual reach of the HIRAID^®^ implementation education program

Cluster	1	2	3	4	Total
Intructor (sites)	4 (3)	8 (6)	1 (1)	2 (1)	15 (11)
Instructors	34	46	25	40	145
Provider courses and number of sites	48/10 sites (100%)	34/ 9 sites (75%)	72/ 4 sites (100%)	56/ 3 sites (100%)	210 / 26 (90%)
Any education at 12 weeks	97% (181)	87% (334)	96% (379)	88% (361)	91% (1255)
All education at 12 weeks	71% (133)	79% (304)	94% (370)	77% (316)	82% (1123)

### Effectiveness

Of the staff who completed the post implementation survey, most (78.1%, *n* = 419) felt HIRAID^®^ helped to “teach new emergency nurses how to assess and manage patients”. This fell short of our target > 80% of positive participant opinion on the usefulness of HIRAID^®^. There was no significant variance across clusters. More than two thirds [72.1% (*n* = 385)] reported they thought about the HIRAID^®^ steps as they do a patient assessment most or all the time. More than two thirds (68.5%, *n* = 366) felt HIRAID^®^ helped them remember to do all parts of a full assessment when needed. The metropolitan cluster with the youngest and least clinically experienced workforce had the largest proportion of those reporting (86.3%, *n* = 138, *p* < 0.0001) they think about the HIRAID^®^ steps while doing a patient assessment and that HIRAID^®^ helped them in clinical practice (85.6%, *n* = 137, *p* < 0.0001) (Supplementary File 3).

There were four themes generated from 721 codes provided in participant free text comments explaining why they did or did not feel HIRAID^®^ supported patient assessment, particularly those new to emergency nursing as a tool that (i) Prompts and provides structure for consistency (ii) enabled more consistent identification of potential problems and communication, (iii) impacts learning, education and training needs; and (iv) impacts current work practice (Table 5).

**Table 5:** Content analysis of nursing survey responses around HIRAID^®^ use, subthemes and exemplar quotes

Why do or don't you use the HIRAID documentation templates?	149 Codes
Quality care and documentation	**83**
*The structure*	***35***
*Consistency and continuity of documentation*	***23***
*Documentation is easier, especially with prompts*	***13***
*Because it is mandatory*	***12***
▪ *“I use them all of the time I think it gives a great summary of the patient from the initial presentation right through to the assessment and plan” (Participant 69 Cluster 1)* ▪ *“Because I am supposed to. I did have a previous template that I felt was sufficient, but best not to rock the boat” (Participant 327 Cluster 2)”*	
Prefer own or old ways of documenting	**33**
*Takes the clinical thinking skills away from nursing staff*	***1***
*Prefer my own way*	***13***
▪ *“what I witness is new / junior diligently filling out hiraid while not attending to the patient's immediate needs. I think it's helpful to have a good template to help junior nurses organise their notes and hopefully direct their care, but the template alone is not going to change practice. What we really need is safe staffing and skill mix so that senior nurses have the time to model and teach excellent assessment, and educators that work and teach "at the elbow" where the learning happens” (Participant 358, Cluster 2)*	
Not appropriate for the clinical situation	**19**
▪ *“If in resus, to many things changing quickly so a note works better. Hi raid can then come later” (Participant 572, Cluster 4)* ▪ *“not every presentation needs an assessment eg. a splinter in a finger.” (Participant 102 Cluster 1)*	
Prioritising direct clinical care	**33**
*Workload and patient acuity*	***22***
*Not enough staff*	***11***
▪ *“We don't have the time! 1 nurse and up to 10 patients It's often IMPOSSIBLE” (Participant 47 Cluster 1)*	
**Why does/does not HIRAID**^**®**^ **support nurses to assess and manage ED patients**	**721 Codes**
Prompts and structure for consistency	391
*Prompts help assessment and reminds*	*192*
*Offers structure and guidance*	*83*
*Takes too long*	*38*
*Reduces anxiety*	*9*
*Comprehensive and consistent*	*69*
▪ *“HIRAID doesn't leave room for forgetting / missing parts of an assessment. It is very busy in ED and sometimes I will forget to assess something in my re-assessment of my patient, and when I begin documenting, the HIRAID template reminds me to go back to the patient and assess what I missed” (participant 149, Cluster 3)* ▪ *“It does prompt me to ask questions of the patient when I am documenting, especially when it is busy, or the patient is very complex, and I am documenting in retrospect” (participant 67, Cluster 1)* ▪ *“HIRAID in a way breaks down doing an assessment. I know I initially got overwhelmed with trying to gather information, but when HIRAID was introduced it simplified it for me” (participant 175, Cluster 4)*	
Structure enables consistent identification of potential problems and communication	187
*Higher quality assessment and communication*	*65*
*Quality of documentation and assessment*	*91*
*Documentation improves assessment quality and communication*	*31*
▪ *“Promotes systematic assessment and good documentation. Especially important at our ED as our ICU is 2 h away by road. HIRAID is a valuable tool for communicating with distant sites” (Participant 24 Cluster 1)* ▪ *“Helps promote BTF and identification of potential issues for the pt that a new may nurse may not have been aware off or experienced before” (Participant 49 Cluster 1)* ▪ *“Hiraid helps to determine the severity of pt's clinical presentation, helps to identify red flags and to do focused assessment based on their situation” (Participant 155, Cluster 3)*	
Impacts learning, education and training needs	45
*HIRAID is a structure, nurses still need to build knowledge through other sources*	
▪ *“What I witness is new / junior diligently filling out hiraid while not attending to the patient's immediate needs. I think it's helpful to have a good template to help junior nurses organise their notes and hopefully direct their care, but the template alone is not going to change practice. What we really need is safe staffing and skill mix so that senior nurses have the time to model and teach excellent assessment, and educators that work and teach "at the elbow" where the learning happens” (Participant 358, Cluster 2)* ▪ *“I agree the HIRAID does help junior nurses to think about their assessment and interventions. I don't think it "teaches"—that is the job of the educator and other more senior nurses” (Participant 324, Cluster 2)*	
Impacts current work practice	98
*Nothing new and the process is time consuming*	27
*Document system and structure takes too long*	71
▪ *“Being an experienced nurse I am used to my own thorough documentation. It feels as if we're being spoon-fed. l, more appropriate for entry level nurses” (Participant 169, Cluster 3)* ▪ *“Spending so much time complete this compulsory structure, takes time away from the patient, looking at the patient. Makes pt's care less direct and personal”. (Participant 63 Cluster 1)*	

### Adoption

Adoption of the intervention was extremely high at the setting and individual levels. The overall site adoption of the intervention was 100%. At least one Instructor from each site attended Instructor training.

**Fig. 2:**
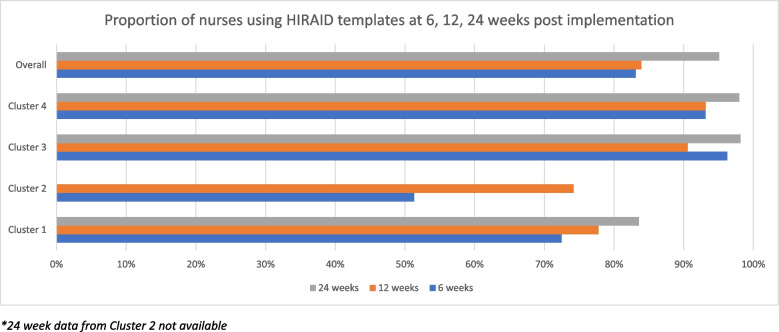
Proportion of nurses using HIRAID templates at 6, 12, 24 weeks post implementation. *24 week data from Cluster 2 not available

At the individual level, overall adoption was high as evidenced by nurse use of the HIRAID^®^ documentation templates. Six weeks post implementation, three initial consecutive nursing entry records of every nurse on shift (*n* = 711) over a 7-day period were audited, 83% demonstrated appropriate use of the HIRAID^®^ documentation templates when indicated at least once. This proportion remained consistent at 12 weeks, 84% of 989 nurses appropriately used the templates (Fig. 2). Uptake at the metropolitan sites was much higher than the rural regional sites (95% vs 76%). Of nurses who completed the survey six months post implementation, 88.6% (*n* = 473) indicated they use the HIRAID^®^ documentation templates most or all the time for their initial documentation. This ranged from 82.5% at one of the rural clusters to 92.6% at the other rural cluster.

There were four themes generated from 149 comments provided by survey respondents in response to questions asking why they did or did not use the HIRAID^®^ documentation templates. The most frequent reason was that a standardised approach with prompts enabled more comprehensive care, consistency of documentation, and reduced cognitive load (Table 5).

### Implementation quality: Dose

Of the 21 BCTs, 100% were used. Dose was high, as assessed through the number and proportion of instructor and provider courses delivered at each cluster and site within the proposed timeline. At a setting level, there were 15 Instructor courses delivered at 11 sites by one of the HIRAID^®^ Chief investigators across all clusters with staff from all sites (100% dose). Then, these Instructors delivered 210 provider courses at 90% (26/29) of sites within the study protocol timeline and study budget. Feedback from 104 of the 145 Instructor course attendees was that the learning outcomes were clearly presented mean (SD) (8.7 (± 1.6), and the course was engaging 9.2 (± 1.4). Respondents indicated they were clear on how to apply the HIRAID^®^ framework in clinical practice 9 (± 1.4) and had a good understanding of their role as a HIRAID^®^ Instructor 8.9 (± 1.4) and felt confident in their ability to deliver the training 8.8 (± 1.3). These high levels of confidence and understanding were consistent across sites and supported by more than 60 free text comments (Table 6).

“Great to hear straight from horse’s mouth. Fantastic tool and as a site who hasn't got it implemented, I feel we have the tools to get started with implementation (Cluster 3)”

“It was fabulous! I can't wait to implement or be involved in the implementation of HIRAID in ED. Very interesting learning the history of HIRAID and understanding the research behind it. So cool! (Cluster 3)”

“It was really well delivered. Just the right amount of laughs throughout (Cluster 4).

“Best "train the trainer" I’ve done. I actually feel I would be able to deliver this education properly (Cluster 2)”

**Table 6:** Behaviour change techniques, mode of delivery and use in HIRAID^®^ implementation

*Behaviour change techniques* and mechanism of action (MOA)	Mode of delivery	Measure / Indicator	Degree of modification	Qualitative Evidence Nurse survey content analysis Instructor survey comments
*5.1 Information on health consequences* Knowledge Belief about consequences	Information on evidence-based health consequences of using HIRAID^®^ including, decreased adverse events in instructor and participant training - Participant workbook - Instructor manual - eLearning module - Face to face training (instructor/participant)	100% sites had instructor 100% sites used training 91% nurses (*n* = 1255) did education	Minor Face to face instructor training became hybrid at small sites in Cluster 2 due to COVID pandemic and catastrophic flooding 12% of instructors modified face to face training to use paper version or modify a slide to localise context	Instructor survey: *When time was limited I used the printed version of the presentation and did quick on the go education. This sometimes included starting and stopping the presentation to deal with ward requirements (Cluster 1)* Nurse survey: Prompts and structure guides enables consistent, comprehensive assessment and appropriate prioritisation (*n* = 391)
*4.1 Instruction on how to perform behaviour* Skills	Instruction in applying HIRAID^®^ in all modes of training	100% sites used training 98.6% of instructors explained what HIRAID^®^ was 95% explained requirements 91% of all nurses did education	Nil	Instructor survey: *I provided education to nurses I was working with on the floor during my clinical duties. I was not given any time off the floor to follow up on other staff's progress, access to the training materials, flip cards or pt's notes to perform audits (Cluster 2)* *After attending the in-service and going through the case scenario, most staff understand the constructs of HIRAID related to patient assessment (Cluster 1)*
*6.1 Demonstration of the behaviour* Skills Social /professional role and identity	- Face to face training (instructor/participant) - Interactive workshops -—Instructors use HIRAID^®^ in clinical practice and routine ED education	As above and Instructors worked with novice nurses to complete patient assessment some/all the time (85%)	Nil	Instructor comments: *Due to acuity and high workload. As the champions were still working on the floor with patient loads (due to poor staffing) we weren't always able to support colleagues as much as we would have liked to (Cluster 4)* *We gave it our best and continue to encourage people to use HIRAID in our ED (Cluster 1)*
*8.1 Practice rehearsal* Skills	- demonstrate HIRAID^®^ in interactive, face to face workshops, ask participants to perform reassessment, and give the group feedback	91% of all nurses did education Practice handover and escalation some/all the time 78%	Nil	Instructor comments: *Some staff were on leave and some casual staff hard to capture (Cluster 1)* *I used the HIRAID documentation templates in my clinical documentation of assessment after triage (Cluster 4)*
*8.3 Habit formation* Skills	Adoption of the HIRAID^®^ framework into broader ED education to increase nurse exposure to relevant application of skill and allows for ongoing training	In 100% orientation at all sites	Nil	*It's become a habit (Cluster 2)*
*7.1 Prompts/cues* Environment context and resources	Integrate HIRAID^®^ documentation templates into electronic medical record system to support and reduce cognitive load	All sites, 100%	Nil	Nurse survey: Structure and format of template enabled quality care and documentation (*n* = 83) Structure enables consistent, identification of potential problems and communication (*n* = 187) *The templates make it so much easier to prompt and document (Cluster 3)*
*12.2 Restructuring the social environment* Environment context and resources	- recruit and train local clinical leaders in use of HIRAID^®^ in instructor courses - Clinical supervision - protected time to complete HIRAID^®^ education program - shift huddles using inclusive language provide feedback to staff on behaviour in using HIRAID^®^ from shift/day before - regular stakeholder meetings to discuss implementation readiness and plans, and to troubleshoot problems	Champions at 100% sites 100% sites committed to protected time 77% used comms at staff huddle Steering committees at each cluster	Nil	Nursing survey comments *I use because encouragement from HIRAID champion. Saved templates make documentation easy (Cluster 1)* *We were supported to attend training, although some people attended in their own time (Cluster 3)* *supportive 1: 1 on the spot training and guidance(Cluster 1)*
*11.3 Conserving mental resources* Environment context and resources	- Prompts in documentation templates - Flip cards to support recall framework steps	100% have doc templates, 83% nurses using 77% said got a flip card, 83% of those said they used it to help HIRAID^®^ recall	Nil	Nursing survey comments *Provides structure in high turnover when feeling stressed (Cluster 3)* *It gives structure to the assessment and I find that I don't miss things. Very helpful to take a breath, think through the components of HIRAID, especially when we are busy. (Cluster 1)*
*6.3 Information about others approval* Social influences Optimism	- Email from hospital executives in support of HIRAID^®^ - Ensure floor staff awareness of management support in meetings/huddles - Management, senior staff and influential peer staff support in video highlighting benefits of HIRAID^®^ - Catch phrases to be used by Instructors to assist in reframing the behaviour with messaging to support/ encourage application in practice - Provide information on evidence and development of education program and implementation plan. For example, the implementation plan was developed using behaviour change theory - In interactive workshops, provide examples of poor documentation and how it has improved since using HIRAID^®^ at other sites	100% sites got memos from executive 77% comms at staff huddle 2 of 4 Clusters chose to do video 100% instructor courses did this	Moderate	*thanks for providing such amazing changes to our processes and documentation (Cluster 4)* *I use because the Educators expect us to. (Cluster 3)*
*3.2 Social support (practical)* Social influences	- Support from educators to allow staff to take time to complete necessary assessment and documentation to increase familiarity and competency - Facilitation by HIRAID^®^ team including site visits, and tracking of site education and issues - Daily walk-through ED by core research nurse/ HIRAID^®^ Instructors to monitor, answer questions, assist, praise, feedback - Ongoing support from research team in education, implementation and stakeholder meetings	100% sites had instructors 89.2% champions used HIRAID^®^ CNC (research team) as a support. Only 6.7% used the teams / virtual drop in	Nil	Nursing survey comments *We were given lots of education and support (Cluster 1)* *Instructors all did really well with the material they had. It was also good having a mix of senior nurses, and nurses who had less experience to allow them to provide education with support from others (Cluster 4)*
*6.2 Social Comparison* Optimism	- Demonstrate to staff how implementation has been achieved at other sites in training	100% sites incorporated in training	Nil	
*5.3 Information about social and environmental consequences* Knowledge Belief about consequences	- Provide information about HIRAID^®^ impacts on emergency nursing care, e.g., improved documentation, and self-confidence in the education program	Evidence included in education	Nil	Nursing survey comments *Wonderful instructors! Great too, for ED! Always great to review assessment skills. Thanks for all the research work* *(Cluster 2)* *It will support new nurses once they understand why we do it and how to do it properly (Cluster 3)* *I use them all the time I think it gives a great summary of the patient from the initial presentation right through to the assessment and plan (Cluster 1)*
*1.4 Action planning* Belief about consequences	- Implementation plan for each site, including who is making the changes, and timeframes and milestones, and progress measures	100% sites	Nil	Managed by research team
*13.2 Framing / reframing* Social /professional role and identity	- Ensure experienced staff understand that it does not necessarily change work practice but provides common terminology and approach to assessment in the ED (Education program)	The phrase *“It is what we already do, just standardising it so we can teach new ED nurses and speak the same language*” delivered in 100% instructor and provider courses	Nil	
*15.1 Verbal persuasion about capability* Social /professional role and identity	- Instructors provided feedback at bedside, addressing areas for improvement and acknowledging where done well	97% Instructors answered questions about HIRAID^®^ some or all of the time 84% were able to work with new emergency nurses some or all of the time	Minor	*The educator and champions followed up with staff to encourage use of the tool and support for questions” (Cluster 1)*
*13.1 Identification of self as a role model* Intentions	- Staff self-nominate as Instructors	90% self nominated 10% appointed by manager	Nil	*Upcoming leaders that were looking for a portfolio to build their CV (Cluster 3)* *HIRAID champions were role models in the department and were happy to support staff (Cluster 3)* *there were 2 instructors at our site. I don't feel that the other champion did 50% of the work. She turned up for training and ate the chocolates. (Cluster 1)*
*9.1 Credible source* Social Influences Optimism	- HIRAID^®^ video includes ED nurse leaders from across the district and country as well as ED staff from clusters supporting HIRAID^®^	Video used in all Instructor courses and 93% of Provider courses Instructors were in leadership roles or emerging leaders	Minor	*HIRAID champions were role models in the department (Cluster 3)* *it was wonderful to meet the leader of the project (Cluster 2)* *I got trained from ED's CNCs and CNEs to do HIRAID as part of my job!! (Cluster 3)*
*2.2 Feedback on behaviour* Reinforcement	- Local nurse leaders, managers and Instructors provide regular feedback to staff individually in coaching during patient care. Praise for a job well done and highlight areas to improve - Instructors provide feedback in practice, support application and documentation - Audit conducted 6/12 weeks during implementation—feedback aggregate on use per site	Documentation audits performed for 100% sites Feedback from managers and instructors regarding use of documentation templates 91% of audit results communicated to staff some or all the time	Nil	Nursing survey Use because mandatory (*n* = 12) *i use HIRAID because we're told to, not because I want to (Cluster 2)* *if the staff aren't using it then they're subject to disciplinary action (Cluster 3)*
*2.7 Feedback on outcomes of behaviour* *Reinforcement* *Beliefs about consequences*	- Interactive feedback provided based on responses in interactive case studies in eLearning and interactive workshops	Education program Instructor support	Nil	Instructor survey: *One on one feedback in a small site made this implementation fairly easy. Review of notes also made follow-up and corrections easy to do (SNSW)*
*7.5 Remove aversive stimulus* *Reinforcement*	- Remove old documentation templates	100% sites	Nil	Managed by research team
*10.1 Material incentive* *Reinforcement* *Optimism*	- Monitor uptake through HIRAID^®^ templates, those consistently using and sites with the greatest proportion will have opportunity to receive prizes- varied by site based on local input	Prizes to first sites to complete 80% staff training Thankyou baskets of healthy food to all EDs from the HIRAID^®^ team	Nil	Managed by research team

### Implementation quality: fidelity

All 21 implemented BCTTs were implemented as intended, three with minor modification and one with moderate modification. The instructor courses (setting level) were delivered with extremely high fidelity. The same cohort of four instructors delivered these and other than the planned site-specific modifications, for example, the names of the hospitals or nursing executive, the content was unchanged. The mode of delivery did vary for one Instructor course as two days before training was to begin in one cluster, catastrophic flooding occurred meaning flights to the venue were cancelled, roads were closed, and several staff lost their homes. This required some modification to how the course was delivered (from all face to face, to hybrid face to face / online), but not the content. There was also some variation to the mode of delivery to some participant courses in the same cluster as the second wave of the COVID-19 pandemic was in its peak. Smaller group sizes, mask wearing and a brief move from all face to face, to hybrid face to face / online courses was necessary (Table 6). No additional funding was required to complete implementation.

HIRAID^®^ champions (self-identified staff to feedback and support in practice) were implemented at all sites however their capacity to complete the intended tasks was moderate (49% all the time, 47% sometimes) and varied across clusters (27% at one metro cluster were able to complete intended tasks all the time compared to 67% at the other metro cluster). Instructors felt they were able to explain what HIRAID^®^ was most of the time (98%) but only 60% felt they always had time and 35% sometimes had time to go through documentation templates with staff. Even fewer felt they had time to work with novice nurses to complete a patient assessment using HIRAID^®^ (49% all the time, 35% sometimes) or practice handover and escalation with novice nurses (32% all the time, 46% sometimes). The HIRAID^®^ teaching materials were delivered by those who had attended instructor training 94% of the time and when the provided slide deck was used (67%), it was used as intended (87.8%). The only changes made were to suit local context, for example, inserting local statistics. The rural clusters used the slides about half the time education was delivered (Supplementary file 4).

### Maintenance / Sustainability

At a setting level, at the time of publication (3 years since implementation), 29 EDs have embedded the HIRAID^®^ training program in their new staff orientation program and recommend use of HIRAID^®^ to inform initial patient assessment and management post triage. This is evidenced by correspondence from site educators with whom we have an ongoing relationship. Since completion of the study, site educators track completion of the HIRAID^®^ program by new staff in local learning tracking software used by each health district for nursing education. Updated training materials, such as revisions to workbooks or readings are distributed to the educators and leadership teams for each site. Electronic documentation templates also remain in use, with the ability for each site to modify for context if needed. eLearning site has been updated and we are able to identify the location of staff who have completed the training. At an individual level, 94.8% (*n* = 506) of respondents to the 6-month post implementation survey indicated they used a pre-planned structure to clinical assess their patient and 88.6% (*n* = 473) said they were using the HIRAID^®^ documentation templates to record this. This was supported by 95% (*n* = 621) of nurses at three clusters demonstrating appropriate use of the templates at least once in a 6-month documentation audit.

## Discussion

The implementation of a behaviour-change informed strategy for the emergency nursing framework HIRAID^®^ in rural, regional, and metropolitan Australia was highly successful, achieving 100% adoption and high reach, effectiveness, adoption dose, fidelity, and sustainability. Guided by the RE-AIM framework [23], we used multiple methods to demonstrate that all 21 BCT and mechanisms as determined by the behaviour change wheel process were used to high fidelity, some requiring minor modification for circumstances beyond our control such as the COVID-19 pandemic and catastrophic flooding.

The use of behaviour change theory enabled maximum uptake of our intervention and adherence to intervention fidelity in the complex ED clinical settings where others have failed [10]. Implementation in emergency contexts is challenging with numerous barriers, with high staff turnover, overcrowding and high priorities. High staff turnover often means there are high numbers of non-permanent staff. Non-permanent staff were not included in the evaluation; however, are an essential part of nursing workforce and need consideration. This has since occurred through generation of “cheat sheets” for how to use HIRAID^®^ and local rapid onboarding processes. The implementation plan included strategies for maintenance of the use of HIRAID^®^ embedded into local roles and practices such as education.

Challenges related to COVID-19 and catastrophic flooding added challenges to implementation. Implementing a major project during a pandemic and a natural disaster meant flexibility in design, delivery and expected progress. Adapting to challenges was critical to progress. Though some of the education sessions had to be adapted to online, our experience was that virtual in-services were well attended, and engagement was not compromised. Adapting to challenges while maintaining high fidelity is possible in the ED [46]; however the success or failure of these adaptations is less certain [47]. Using implementation science frameworks to be adaptive to the needs of the sites during implementation and having strong champions and site engagement has been shown to be successful [48]. Our experience was that relationships and effective regular communication with stakeholders were critical components to problem solve and meet local needs, cognisant of impact on personal impacts on staff related to the major events. As the implementation was part of a large funded study, there was access to highly experienced implementation specialists and expert emergency clinician researchers, access to specialists and funding to enable expertise and support would likely contribute to the success of implementation [49].

Our evaluation adds to and challenges the work of Connell et al. [50] around the four of the ten BCTs and Mechanisms of Action reported by a panel of 105 experts as having no definite links. In developing our implementation strategy, we allocated the Mechanisms of Action “Optimism” and “Social Professional Role and Identity” to address barriers related to feelings of a lack of support by management and a lack of time to change practice. Optimism, that is, the confidence that things will happen for the best or that desired goals are attained was effectively linked to two BCTs in this study: (i) *6.3 Information about others approval* was demonstrated through leadership support for implementation, e.g. memo from hospital executives to nursing staff supporting the project, and commitment of work time to complete education. In addition, leadership approval was demonstrated in a video for viewing in the training program featured senior staff and relevant team members. (ii) *6.2 Social Comparison* was used to reassure staff about the change and provide evidence to staff how implementation was achieved at other sites in all training.

“Social Professional Role and Identity” was linked to three BCTs (i) *6.1 demonstration of the behaviour* by having instructors from other sites attend train the trainer sessions at other clusters demonstrating and modelling behaviour (ii)*13.2 Framing / reframing* to ensure experienced staff understand that the HIRAID^®^ intervention does not necessarily change work practice but provides common terminology and approach to assessment in the ED. This was incorporated throughout the education program and, (iii) *15.1 Verbal persuasion about capability*. Instructors developed phrases to support/ encourage staff use of HIRAID^®^- delivered and codesigned in the Instructor education program that were then used as part of implementation.

Two BCTs *Action Planning (1.4)* and *Credible Source (9.1)* were linked to three mechanisms of action. *Action Planning (1.4)* was successfully linked to the mechanisms of action “Belief about consequences” to address the barrier “a lack of belief that change will happen”. Action planning was used to operationalise an implementation plan for each site, including who is making the changes, timeframes, milestones and progress measures. This demonstrated to staff that there was accountability and expectation that change would occur. “Credible Source (9.1)” was linked to the mechanisms of action *Goals* to facilitate the enabler “evidence that implementation of the intervention will help do what is best for patient care” and *Social Influences* to address the barrier “lack of support” through an email from executive outline their support of HIRAID^®^ as evidenced by allocation of non-clinical time to attend training.

### Research and practical implications

The generation of robust evidence for implementation strategy effectiveness and measurement is in its infancy, particularly in the emergency setting [23], however, this research has advanced knowledge in this field, for example, by highlighting the importance of social influence, professional identity, reframing and verbal persuasion [51]. These were achieved effectively in this study using site champions [52]; however, would unlikely be effective as a strategy alone [53]. Targeted contextual barrier assessment and identification of relevant strategies were successful in this implementation study, which may be the key until we have further evidence on specific strategies. Planning without use of implementation science frameworks is not adequate in the emergency context [54] and consideration to enabling and engaging front line clinicians is crucial.

In this study, we used the RE-AIM framework to guide the evaluation of the implementation, finding it appropriate for our needs in this study. The evaluation plan was developed prior to implementation of the study and developed benchmarks; however, some benchmarks were arbitrarily selected as others have identified [55]. There is limited evidence in the implementation fidelity literature for setting benchmarks, much of which focuses on adaptations. Implementation research guidelines on levels of fidelity, adaptations and sustainability in the acute care and emergency setting should be developed to inform future research.

This study provided evidence that a novel framework for emergency nursing can be adapted to context and delivered as intended yielding a high reach. The results of this study informed changes to the implementation plan and the development of an implementation toolkit to support implementation spread and scale-up at new sites wanting to implement HIRAID^®^. Since implementation at the study sites, HIRAID^®^ has been implemented using our implementation plan at more than 50 EDs in Australia, and to varying degrees in Fiji, Canada, USA, China and Singapore through their emergency nursing associations (see Supplementary material 5). We are working our study partners, the Australian College of Nursing and the Australian Commission for Quality and Safety in Health care to further upscale locally.

### Methodological considerations and limitations

There were some limitations to this study. Our sites represented rural, regional, and metropolitan settings however may still not be generalisable to all contexts. Our implementation strategy should not be used without examination and consideration for the proposed context. Documentation audits may not give a true indication of staff members using HIRAID^®^ framework in their assessments. The surveys were developed based on responses needed to address the RE-AIM criteria, however, there were no standardised questions available. The qualitative questions were limited to questions about the use of the tool rather than evaluation of the process, which may limit the interpretation of some of the results. There was some delay between the implementation and the surveys which may have resulted in participants misremembering some answers. We did not conduct documentation audits for non-permanent staff as they were less frequently on shift, allocated shifts through different rostering systems and not formally part of implementation however their contribution to emergency care is crucial and their training and education is important to consider in further studies in emergency contexts. The next phase of this program of research is to evaluate the impact of the implementation of HIRAID^®^ on patient and health service outcomes, including cost–benefit to determine the economic soundness of the intervention.

## Conclusion

The implementation of a behaviour-change informed strategy for the emergency nursing framework HIRAID^®^ in rural, regional, and metropolitan Australia was highly successful, achieving 100% setting adoption with high reach, dose, fidelity, and sustainability up to 3 years post implementation. The use of behaviour change theory enabled maximum uptake of our intervention and adherence to intervention fidelity in complex ED clinical settings. We made some modifications to the original implementation strategy in response to major incidents. The project was delivered within the study protocol timeline and study budget. Future upscaling can be informed by this evidenced-based, reliable strategy.

## Supplementary Information

Supplementary Material 1.

## Data Availability

The original datasets generated and analytic data sets for this study are not publicly available because of HREC requirements. De-identified and aggregated data sets may be available on application to the corresponding author and subject to the HREC approval.

## Abbreviations

HIRAID^**®**^: History including Infection risk, Red flags, Assessment, Interventions, Diagnostics, communication, and reassessment
BCT: Behaviour change technique
BCTT: Behaviour change technique taxonomy
ED: Emergency department
km^2^: Kilometre
LHD: Local health district
SW-Crct: Step–wedge cluster randomised control trial
RE-AIM: Reach, Effectiveness, Adoption, Implementation (dose/fidelity) and Maintenance / Sustainability
REDCap™: Research Electronic Data Capture
AUD: Australian Dollar
SPSS: Statistics Package for Social Sciences

## References

[1] Curtis K, Brysiewicz P, Shaban RZ, Fry M, Considine J, Gamboa FEA, et al. Nurses responding to the World Health Organization (WHO) priority for emergency care systems for universal health coverage. Int Emerg Nurs. 2020;50. 10.1016/j.ienj.2020.100876 PMC7188622 32446745

[2] Munroe B, Curtis K, Margerat M, Strachan L, Buckley T. HIRAID: An evidence-informed emergency nursing assessment framework. Aust Emerg Nurs J. 2015;18(2):83–97. 10.1016/j.aenj.2015.02.001 25863915

[3] Curtis K, Munroe B, Van C, Elphick TL. The implementation and usability of HIRAID, a structured approach to emergency nursing assessment. Australas Emerg Care. 2020;23(1):62–70. 10.1016/j.auec.2019.10.001. 31699613 10.1016/j.auec.2019.10.001

[4] Curtis K, Munroe B, Fry M, Considine J, Tuala E, Watts M, et al. The implementation of an emergency nursing framework (HIRAID) reduces patient deterioration: A multi-centre quasi-experimental study. Int Emerg Nurs. 2021;56: 100976. 33882400 10.1016/j.ienj.2021.100976

[5] Munroe B, Curtis K, Considine J, Buckley T. The impact structured patient assessment frameworks have on patient care: an integrative review. J Clin Nurs. 2013;22(21–22):2991–3005. 23656285 10.1111/jocn.12226

[6] Munroe B, Curtis K, Fry M, Shaban RZ, Moules P, Elphick TL, et al. Increasing accuracy in documentation through the application of a structured emergency nursing framework: A multisite quasi-experimental study. J Clin Nurs. 2022;31(19–20):2874–85. 10.1111/jocn.16115. 34791742 10.1111/jocn.16115

[7] Curtis K, Sivabalan P, Bedford DS, Considine J, D’Amato A, Shepherd N, et al. Implementation of a structured emergency nursing framework results in significant cost benefit. BMC Health Serv Res. 2021;21(1):1318. 34886873 10.1186/s12913-021-07326-y PMC8655998

[8] Shelton RC, Cooper BR, Stirman SW. The Sustainability of Evidence-Based Interventions and Practices in Public Health and Health Care. Ann Rev Public Health. 2018;39(Volume 39, 2018):55–76. 10.1146/annurev-publhealth-040617-014731 29328872

[9] Middleton S, Dale S, Cheung NW, Cadilhac DA, Grimshaw JM, Levi C, et al. Nurse-initiated acute stroke care in emergency departments. Stroke. 2019;50(6):1346–55. 31092163 10.1161/STROKEAHA.118.020701

[10] McInnes E, Dale S, Craig L, Phillips R, Fasugba O, Schadewaldt V, et al. Process evaluation of an implementation trial to improve the triage, treatment and transfer of stroke patients in emergency departments (T3 trial): a qualitative study. Implement Sci. 2020;15(1):99. 33148343 10.1186/s13012-020-01057-0 PMC7640433

[11] Weiner SG, Hoppe JA. Accelerating Practice Change in the Emergency Department. JAMA Network Open. 2023;6(4):e235453-e. 10.1001/jamanetworkopen.2023.5453 37017976

[12] Rogers L, De Brún A, Birken SA, Davies C, McAuliffe E. The micropolitics of implementation; a qualitative study exploring the impact of power, authority, and influence when implementing change in healthcare teams. BMC Health Serv Res. 2020;20(1):1059. 33228702 10.1186/s12913-020-05905-z PMC7684932

[13] Curtis K, Fry M, Shaban RZ, Considine J. Translating research findings to clinical nursing practice. J Clin Nurs. 2016;26:862–72. 27649522 10.1111/jocn.13586 PMC5396371

[14] Lawrie L, Duncan EM, Jansen JO, Campbell MK, Brunsdon D, Skea Z, et al. Behavioural optimisation to address trial conduct challenges: case study in the UK-REBOA trial. Trials. 2022;23(1):398. 35550599 10.1186/s13063-022-06341-6 PMC9097042

[15] Curtis K, Jansen P, Mains M, O’Hare A, Scotcher B, Alcorn D, et al. Rapid development and implementation of a behaviour change strategy to improve COVID-19 personal protective equipment use in a regional Australian emergency department. Aust Emerg Care. 2022;25(4):273–82. 10.1016/j.auec.2022.01.004 PMC8802564 35123929

[16] Curtis K, Kourouche S, Asha S, Considine J, Fry M, Middleton S, et al. Impact of a care bundle for patients with blunt chest injury (ChIP): A multicentre controlled implementation evaluation. PLoS ONE. 2021;16(10): e0256027. 34618825 10.1371/journal.pone.0256027 PMC8496821

[17] Newlands R, Duncan E, Presseau J, Treweek S, Lawrie L, Bower P, et al. Why trials lose participants: A multitrial investigation of participants’ perspectives using the theoretical domains framework. J Clin Epidemiol. 2021;137:1–13. 33727134 10.1016/j.jclinepi.2021.03.007

[18] Curtis K, Fry M, Kourouche S, Kennedy B, Considine J, Alkhouri H, et al. Implementation evaluation of an evidence-based emergency nursing framework (HIRAID): study protocol for a step-wedge randomised control trial. BMJ Open. 2023;13(1): e067022. 36653054 10.1136/bmjopen-2022-067022 PMC9853264

[19] Michie S, van Stralen MM, West R. The behaviour change wheel: A new method for characterising and designing behaviour change interventions. Implement Sci. 2011;6:42. 21513547 10.1186/1748-5908-6-42 PMC3096582

[20] Michie S, Richardson M, Johnston M, Abraham C, Francis J, Hardeman W, et al. The behavior change technique taxonomy (v1) of 93 hierarchically clustered techniques: building an international consensus for the reporting of behavior change interventions. Ann Behav Med. 2013;46(1):81–95. 23512568 10.1007/s12160-013-9486-6

[21] McGee D, Lorencatto F, Matvienko-Sikar K, Toomey E. Surveying knowledge, practice and attitudes towards intervention fidelity within trials of complex healthcare interventions. Trials. 2018;19(1):504. 30231917 10.1186/s13063-018-2838-6 PMC6147031

[22] Slaughter SE, Hill JN, Snelgrove-Clarke E. What is the extent and quality of documentation and reporting of fidelity to implementation strategies: A scoping review. Implement Sci. 2015;10(1):129. 26345357 10.1186/s13012-015-0320-3 PMC4562107

[23] Guerin RJ, Glasgow RE, Tyler A, Rabin BA, Huebschmann AG. Methods to improve the translation of evidence-based interventions: A primer on dissemination and implementation science for occupational safety and health researchers and practitioners. Saf Sci. 2022;152: 105763. 37854304 10.1016/j.ssci.2022.105763 PMC10583726

[24] Proctor E, Silmere H, Raghavan R, Hovmand P, Aarons G, Bunger A, et al. Outcomes for implementation research: conceptual distinctions, measurement challenges, and research agenda. Adm Policy Ment Health. 2011;38(2):65–76. 20957426 10.1007/s10488-010-0319-7 PMC3068522

[25] Curran GM, Bauer M, Mittman B, Pyne JM, Stetler C. Effectiveness-implementation hybrid designs: combining elements of clinical effectiveness and implementation research to enhance public health impact. Med Care. 2012;50(3):217–26. 22310560 10.1097/MLR.0b013e3182408812 PMC3731143

[26] Wolfenden L, Foy R, Presseau J, Grimshaw JM, Ivers NM, Powell BJ, et al. Designing and undertaking randomised implementation trials: guide for researchers. BMJ. 2021;372: m3721. 33461967 10.1136/bmj.m3721 PMC7812444

[27] Glasgow RE, Harden SM, Gaglio B, Rabin B, Smith ML, Porter GC, et al. RE-AIM planning and evaluation framework: adapting to new science and practice with a 20-year review. Front Public Health. 2019;7:64. 30984733 10.3389/fpubh.2019.00064 PMC6450067

[28] Evidence-Based Cancer Control Programs (EBCCP). RE-AIM Scoring Instrument: National Cancer Institute; 2013 [Available from: https://ebccp.cancercontrol.cancer.gov/reAimCriteria.do#.

[29] Harden SM, et al. Fidelity to and comparative results across behavioral interventions evaluated through the RE-AIM framework: a systematic review. Syst Rev. 2015;4(1):155. 26547687 10.1186/s13643-015-0141-0 PMC4637141

[30] Glasgow RE, Vogt TM, Boles SM. Evaluating the public health impact of health promotion interventions: the RE-AIM framework. Am J Public Health. 1999;89(9):1322–7. 10474547 10.2105/ajph.89.9.1322 PMC1508772

[31] National Health and Medical Research Council (NHMRC). Australian Code for the Responsible Conduct of Research. 2018.

[32] Pinnock H, Barwick M, Carpenter CR, Eldridge S, Grandes G, Griffiths CJ, et al. Standards for Reporting Implementation Studies (StaRI) Statement. BMJ. 2017;356: i6795. 28264797 10.1136/bmj.i6795 PMC5421438

[33] Hemming K, Taljaard M, McKenzie JE, Hooper R, Copas A, Thompson JA, et al. Reporting of stepped wedge cluster randomised trials: extension of the CONSORT 2010 statement with explanation and elaboration. BMJ. 2018;363:k1614. 10.1136/bmj.k1614. 10.1136/bmj.k1614 PMC6225589 30413417

[34] Australia New Zealand Clinical Trials Registry. The impact of the HIRAID emergency nursing program on patient and hospital outcomes - ACTRN12621001456842 2021 [updated 16/11/2023. Available from: https://www.anzctr.org.au/Trial/Registration/TrialReview.aspx?id=382551&isReview=true.

[35] Kourouche S, Curtis K, Munroe B, Watts M, Balzer S, Buckley T. Implementation strategy fidelity evaluation for a multidisciplinary Chest Injury Protocol (ChIP). Implement Sci Commun. 2021;2(1):86. 34376254 10.1186/s43058-021-00189-8 PMC8353870

[36] Harris PA, Taylor R, Thielke R, Payne J, Gonzalez N, Conde JG. Research electronic data capture (REDCap)—A metadata-driven methodology and workflow process for providing translational research informatics support. J Biomed Inform. 2009;42(2):377–81. 18929686 10.1016/j.jbi.2008.08.010 PMC2700030

[37] Abdelazeem B, Hamdallah A, Rizk MA, Abbas KS, El-Shahat NA, Manasrah N, et al. Does usage of monetary incentive impact the involvement in surveys? A systematic review and meta-analysis of 46 randomized controlled trials. PLoS ONE. 2023;18(1): e0279128. 36649255 10.1371/journal.pone.0279128 PMC9844858

[38] IBM Corp. SPSS Statistics for Windows. 22.0 ed. New York, United States: IBM Armonk; 2013.

[39] QSR International Pty Ltd. NVivo qualitative data analysis Software. 10 ed. 2012.

[40] Graneheim UH, Lundman B. Qualitative content analysis in nursing research: concepts, procedures and measures to achieve trustworthiness. Nurse Educ Today. 2004;24(2):105–12. 14769454 10.1016/j.nedt.2003.10.001

[41] Graneheim UH, Lindgren B-M, Lundman B. Methodological challenges in qualitative content analysis: A discussion paper. Nurse Educ Today. 2017;56:29–34. 28651100 10.1016/j.nedt.2017.06.002

[42] Fetters MD, Curry LA, Creswell JW. Achieving integration in mixed methods designs-principles and practices. Health Serv Res. 2013;48(6 Pt 2):2134–56. 24279835 10.1111/1475-6773.12117 PMC4097839

[43] Wickersham K, Colbert A, Caruthers D, Tamres L, Martino A, Erlen JA. Assessing Fidelity to an Intervention in a Randomized Controlled Trial to Improve Medication Adherence. Nurs Res. 2011;60(4):264–9. 21677597 10.1097/NNR.0b013e318221b6e6 PMC3138209

[44] Perepletchikova F, Kazdin AE. Treatment integrity and therapeutic change: Issues and research recommendations. Clin Psychol Sci Pract. 2005;12(4):365–83.

[45] Gregory J. McHugo PD, Robert E. Drake MD, Ph.D. ,, Rob Whitley PD, Gary R. Bond PD, Kikuko Campbell MPH, M.A. ,, Charles A. Rapp PD, et al. Fidelity Outcomes in the National Implementing Evidence-Based Practices Project. Psychiatric Serv. 2007;58(10):1279–84. 10.1176/ps.2007.58.10.1279 17914003

[46] Murray E, Roosevelt GE, Vogel JA. Screening for health-related social needs in the emergency department: Adaptability and fidelity during the COVID-19 pandemic. Am J Emerg Med. 2022;54:323.e1-.e4. 10.1016/j.ajem.2021.09.071 PMC8492605 34654599

[47] Hall JN, Ackery AD, Dainty KN, Gill PS, Lim R, Masood S, et al. Designs, facilitators, barriers, and lessons learned during the implementation of emergency department led virtual urgent care programs in Ontario, Canada. Front Digital Health. 2022;4:946734. 10.3389/fdgth.2022.946734. eCollection 2022. 10.3389/fdgth.2022.946734 PMC9448924 36093385

[48] Southerland LT, Gulker P, Van Fossen J, Rine-Haghiri L, Caterino JM, Mion LC, et al. Implementation of geriatric screening in the emergency department using the Consolidated Framework for Implementation Research. Acad Emerg Med. 2023;30(11):1117–28. 37449967 10.1111/acem.14776 PMC11195318

[49] Shepherd M, Endacott R, Quinn H. Bridging the gap between research and clinical care: strategies to increase staff awareness and engagement in clinical research. J Res Nurs. 2022;27(1–2):168–81. 35392210 10.1177/17449871211034545 PMC8980567

[50] Connell LE, Carey RN, de Bruin M, Rothman AJ, Johnston M, Kelly MP, Michie S. Links between behavior change techniques and mechanisms of action: an expert consensus study. Ann Behav Med. 2019;53(8):708–20. 30452535 10.1093/abm/kay082 PMC6636885

[51] Kharel P, Zadro JR, Wong G, Rojanabenjawong K, Traeger A, Linklater J, Maher CG. Effectiveness of implementation strategies for increasing clinicians’ use of five validated imaging decision rules for musculoskeletal injuries: a systematic review. BMC Emerg Med. 2024;24(1):84. 38760697 10.1186/s12873-024-00996-x PMC11100091

[52] Damush TM, Miech EJ, Rattray NA, Homoya B, Penney LS, Cheatham A, et al. Implementation evaluation of a complex intervention to improve timeliness of care for veterans with transient ischemic attack. J Gen Intern Med. 2021;36(2):322–32. 33145694 10.1007/s11606-020-06100-w PMC7878645

[53] Penney LS, Moreau JL, Miake-Lye I, Lewis D, D’Amico A, Lee K, et al. Spreading the Veterans Health Administration’s emergency department rapid access clinics (ED-RAC) innovation: Role of champions and local contexts. Healthcare. 2021;9(2): 100516. 33384257 10.1016/j.hjdsi.2020.100516

[54] Liu J, Ponzer S, Farrokhnia N, Masiello I. Evaluation of interprofessional teamwork modules implementation in an emergency department – A mixed-methods case study of implementation fidelity. BMC Health Serv Res. 2021;21(1):853. 34419021 10.1186/s12913-021-06822-5 PMC8380355

[55] Cornell PY, Hua CL, Halladay CW, Halaszynski J, Harmon A, Koget J, Silva JW. Benefits and challenges in the use of RE-AIM for evaluation of a national social work staffing program in the veterans health administration. Front Health Serv. 2023;3:1225829. 38034078 10.3389/frhs.2023.1225829 PMC10687433

