# Correction: Successful and sustained implementation of a behaviour-change informed strategy for emergency nurses: a multicentre implementation evaluation

**DOI:** 10.1186/s13012-024-01405-4

**Published:** 2024-11-01

**Authors:** Kate Curtis, Belinda Kennedy, Julie Considine, Margaret Murphy, Mary K. Lam, Christina Aggar, Margaret Fry, Ramon Z. Shaban, Sarah Kourouche

**Affiliations:** 1 Susan Wakil School of Nursing and Midwifery, Faculty of Medicine and Health, RC Mills Building, University of Sydney, Camperdown, NSW 2006 Australia; 2 Emergency Services, Illawarra Shoalhaven Local Health District, Wollongong Hospital, Crown St, Wollongong, NSW Australia; 3 School of Nursing and Midwifery and Centre for Quality and Patient Safety Experience in the Institute for Health Transformation, Deakin University, Geelong, VIC Australia; 4 Centre for Quality and Patient Safety Research – Eastern Health Partnership, Box Hill, VIC Australia; 5 Western Sydney Local Health District, North Parramatta, NSW 2141 Australia; 6 School of Health and Biomedical Sciences, RMIT University, Melbourne, VIC Australia; 7 Northern NSW Local Health District, Southern Cross University, Lismore, Australia; 8 Sydney Faculty of Health, University of Technology, Ultimo, NSW Australia; 9 Northern Sydney Local Health District, St Leonards, NSW Australia; 10 Sydney Infectious Diseases Institute, Faculty of Medicine and Health, The University of Sydney, Camperdown, NSW 2006 Australia; 11 Research and Education Network, Western Sydney Local Health District, Westmead, NSW 2145 Australia; 12 New South Wales Biocontainment Centre, Western Sydney Local Health District, Westmead, NSW 2145 Australia

**Correction** **:** **Implement Sci 19, 54 (2024)**

**https://doi.org/10.1186/s13012-024-01383-7**

Following the publication of the Original Article [1], the authors identified errors in Table 2. In the Table 2, Cluster 3, commencement date should read **08/08/2022** instead of 08/08/2023.

The Original Article has been corrected.
