# Supplementary material for: Successful and sustained implementation of a behaviour-change informed strategy for emergency nurses: a multicentre implementation evaluation
Source: Implement Sci. 2024 Jul 29;19:54. doi: 10.1186/s13012-024-01383-7 (PMC11285323; doi:10.1186/s13012-024-01383-7)

## Supplementary material 1: Development of the HIRAID® Implementation Strategy


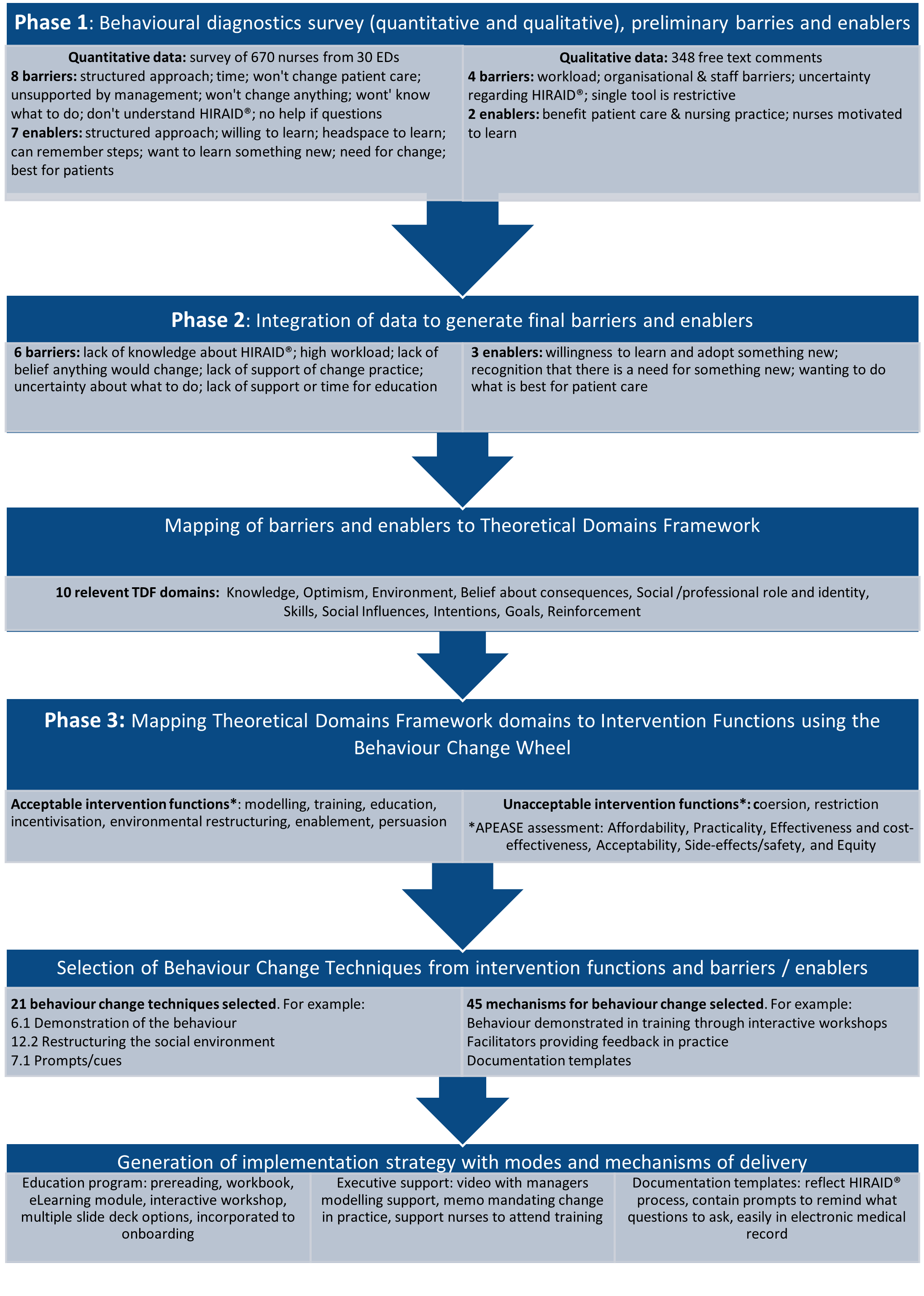


## Supplementary material 2: Nursing staff survey post HIRAID intervention

Thank you for considering participating to provide your perspective on nursing assessment when caring for patient presenting to the emergency department or multipurpose service.

Your feedback is important to both inform the implementation and evaluation of HIRAID (**H**istory and infection risk, **R**ed flags, **A**ssessment, **I**nterventions, **D**iagnostics, communication and reassessment), a new model of nursing care into your work environment. All nursing staff permanently employed at the time of the study will be invited prior to, and following, the implementation of the HIRAID framework.

This survey should take no longer than 15 minutes.

On completion of the survey, you will have the opportunity to provide your contact details for the purpose of a $20 gift voucher, as acknowledgement for your time. The details you provide will not be linked to your survey, your responses will remain confidential, you cannot be identified.

If you have any questions related to this research study, please contact one of the investigators below prior to proceeding.

If you have not read the participant information, a copy can be accessed **here** (*link to Appendix 13 Nursing staff participant information)*.

Please confirm you have read and understood the participant information provided to you

**Thank you please continue to the survey**

### Instrument 1: Demographics (pre and post)

Current position (Select one):

[ ] RN

[ ] EEN

[ ] NUM/ CNC/ CNS/ NP

[ ] other (specify) ____________

How many years have you worked as nurse?

How many years have you worked in the **emergency department**?

Please tick your highest level of post graduate nursing qualification:

[ ] None

[ ] Graduate Certificate

[ ] Graduate Diploma

[ ] Masters

Other:_________________

What areas of the ED do you work (select all applicable)?

[ ] General acute area

[ ] Fast track

[ ] Paediatrics

[ ] Triage

[ ] Resuscitation room

[ ] Other __________________________

Please select the (*insert relevant LHD*) facility that you **currently** work at**:**

*Provide selection options for all sites within the LHD participating in the study*

The questions below relate to the strategies used with the introduction of the HIRAID framework to your ED. We want to know which ones you received or participated in and your experience of the introduction. Please be honest and provide greater details where the opportunity is provided.

|  | **Item** | **Response** | **Justification** |
| --- | --- | --- | --- |
|  | Did you watch the short video about HIRAID featuring staff from across *[insert LHD]*? | 0, No  1, Yes | Dose – staff views for video in implementation |
|  | Were you given a HIRAID flip card to attach to your hospital ID with the HIRAID framework? | 0, No  1, Yes | Implementation (dose) |
|  | *[logic if flipcard=1]*  Did you connect to your ID? | 0, No  1, Yes |  |
|  | Have you ever used the flip card attached to your ID to help recall of the HIRAID framework? | 1, never  2, seldom  3, Sometimes  4, most of the time  5, always | Participant responsiveness- adoption/ uptake |
|  | Did you receive any HIRAID education from a CNC/ Nurse educator or HIRAID clinical champion? | 0, No  1, Yes  3, Cannot recall | Implementation (dose) |
|  | How was this education delivered? | 1, in-service (group) away from clinical area  2, in-service (group) in clinical area  3, 1:1 away from clinical space  4, 1:1 in clinical area  5, Skype/ remote learning  6, other | Implementation (fidelity) |
|  | [if edu_del =6]  Please explain how the education was delivered | Free text |  |
|  | Any comments related to the HIRAID education you received? | Free text |  |
|  | Do you think about the HIRAID steps as you do a patient assessment in the ED? | 1, never  2, seldom  3, Sometimes  4, most of the time  5, always | Participant responsiveness- nurses engaged in using the intervention |
|  | Do you think HIRAID helps you remember to do all parts of a full assessment when needed? |  | Participant responsiveness |
|  | Please comment | text | Provide greater understanding of the use of the Framework |
|  | Do you use the HIRAID documentation templates in ED for your initial documentation (post triage)? | 1, never  2, seldom  3, Sometimes  4, most of the time  5, always | Implementation (dose) |
|  | What are the reasons why you do not use them?  *(select all that apply)* | 1, takes too long  2, too hard to follow  3, I don’t need to do an assessment on some patients  4, too much detail needed  5, not appropriate for patient presentation  6, I have my own way  7, other | Barriers |
|  | *(Logic docu_no=1-6)*  Please provide further details | Free text |  |
|  | Please explain ‘other’ reason/s for not using the HIRAID template. | Free text |  |
|  | Do you think the HIRAID helps teach new emergency nurse about how to assess and manage patients? | 1, never  2, seldom  3, Sometimes  4, most of the time  5, always | Participant responsiveness |

## Supplementary material 3: Six-month post implementation survey results

| **Component items** | **All Sites** | **1** | **2** | **3** | **4** |  | **P- value** |
| --- | --- | --- | --- | --- | --- | --- | --- |
|  |  | **N (%)** | **N (%)** | **N (%)** | **N (%)** | **Test Statistics** |  |
| **Were you given a HIRAID flip card to attach to your hospital ID with the HIRAID framework?** |  |  |  |  |  | **X2(3)=11.16** | **0.011** |
| No- 0 | 166 (31.1%) | 32 (33%) | 34 (27.9%) | 64 (40%) | 36 (23.2%) |  |  |
| Yes- 1 | 368 (68.9%) | 65 (67%) | 88 (72.1%) | 96 (60%) | 119 (76.8%) |  |  |
| **Have you ever used the flip card attached to your ID to help recall of the HIRAID framework?** |  |  |  |  |  |  |  |
| No- 0 | 69 (18.8%) | 8 (12.3%) | 20 (22.7%) | 21 (21.9%) | 20 (16.8%) |  |  |
| Yes- 1 | 299 (81.3%) | 57 (87.7%) | 68 (77.3%) | 75 (78.1%) | 99 (83.2%) |  |  |
| **flipcard_2** *(if flipcard =1)* |  |  |  |  |  | X2(9)=42.24 | <0.0001 |
| Never - 1 | 121 (32.9%) | 15 (23.1%) | 45 (51.1%) | 27 (28.1%) | 34 (28.6%) |  |  |
| Seldom- 2 | 85 (23.1%) | 17 (26.2%) | 27 (30.7%) | 16 (16.7%) | 25 (21%) |  |  |
| Sometimes- 3 | 104 (28.3%) | 21 (32.3%) | 8 (9.1%) | 29 (30.2%) | 46 (38.7%) |  |  |
| Mostly/Always - 4+5 | 58 (15.8%) | 12 (18.5%) | 8 (9.1%) | 24 (25%) | 14 (11.8%) |  |  |
| **Did you receive any HIRAID education from a CNC/ Nurse educator or HIRAID clinical champion?** |  |  |  |  |  |  |  |
| No- 0 | 17 (17.5%) | 17 (17.5%) |  |  |  |  |  |
| Yes- 1 | 79 (81.4%) | 79 (81.4%) |  |  |  |  |  |
| Cannot recall - 3 | 1 (1%) | 1 (1%) |  |  |  |  |  |
| In-service away clinical - 1 | 98 (19%) | 27 (34.2%) | 71 (58.2%) |  |  |  |  |
| In-service clinical - 2 | 29 (5.6%) | 20 (25.3%) | 9 (7.4%) |  |  |  |  |
| 1:1 away clinical - 3 | 19 (3.7%) | 12 (15.2%) | 7 (5.7%) |  |  |  |  |
| 1:1 in clinical - 4 | 28 (5.4%) | 18 (22.8%) | 10 (8.2%) |  |  |  |  |
| Skype remote - 5 | 23 (4.5%) |  | 16 (13.1%) |  | 7 (4.5%) |  |  |
| Other - 6 | 3 (0.6%) | 2 (2.5%) | 1 (0.8%) |  |  |  |  |
| Did not attend in service -7 | 52 (10.1%) |  | 8 (6.6%) | 15 (9.4%) | 29 (18.7%) |  |  |
| 4 hour face to face- 8 | 179 (34.7%) |  |  | 145 (90.6%) | 34 (21.9%) |  |  |
| 1 hour face to face - 9 | 85 (16.5%) |  |  |  | 85 (54.8%) |  |  |
| **Do you think about the HIRAID steps as you do a patient assessment in the ED?** |  |  |  |  |  | X2(9)=40.22 | <0.0001 |
| Never - 1 | 30 (5.6%) | 2 (2.1%) | 13 (10.7%) | 5 (3.1%) | 10 (6.5%) |  |  |
| Seldom- 2 | 34 (6.4%) | 4 (4.1%) | 14 (11.5%) | 4 (2.5%) | 12 (7.7%) |  |  |
| Sometimes- 3 | 85 (15.9%) | 15 (15.5%) | 21 (17.2%) | 13 (8.1%) | 36 (23.2%) |  |  |
| Mostly/Always - 4+5 | 385 (72.1%) | 76 (78.4%) | 74 (60.7%) | 138 (86.3%) | 97 (62.6%) |  |  |
| **Do you think HIRAID helps you remember to do all parts of a full assessment when needed?** |  |  |  |  |  | X2(9)=60.46 | <0.0001 |
| Never - 1 | 28 (5.2%) | 3 (3.1%) | 14 (11.5%) | 0 (0%) | 11 (7.1%) |  |  |
| Seldom- 2 | 44 (8.2%) | 4 (4.1%) | 20 (16.4%) | 6 (3.8%) | 14 (9%) |  |  |
| Sometimes- 3 | 96 (18%) | 16 (16.5%) | 22 (18%) | 17 (10.6%) | 41 (26.5%) |  |  |
| Mostly/Always - 4+5 | 366 (68.5%) | 74 (76.3%) | 66 (54.1%) | 137 (85.6%) | 89 (57.4%) |  |  |
| **Do you use the HIRAID documentation templates in ED for your initial documentation (post triage)?** |  |  |  |  |  |  |  |
| Never - 1 | 9 (1.7%) | 3 (3.1%) | 1 (0.8%) | 3 (1.9%) | 2 (1.3%) |  |  |
| Seldom- 2 | 10 (1.9%) | 3 (3.1%) | 2 (1.6%) | 2 (1.3%) | 3 (1.9%) |  |  |
| Sometimes- 3 | 42 (7.9%) | 11 (11.3%) | 6 (4.9%) | 8 (5%) | 17 (11%) |  |  |
| Mostly/Always - 4+5 | 473 (88.6%) | 80 (82.5%) | 113 (92.6%) | 147 (91.9%) | 133 (85.8%) |  |  |
| **Do you think the HIRAID helps teach new emergency nurse about how to assess and manage patients?** |  |  |  |  |  |  |  |
| Never - 1 | 9 (1.7%) |  | 6 (4.9%) | 1 (0.6%) | 2 (1.3%) |  |  |
| Seldom- 2 | 17 (3.2%) |  | 10 (8.2%) | 2 (1.3%) | 5 (3.2%) |  |  |
| Sometimes- 3 | 89 (16.7%) | 11 (11.3%) | 24 (19.7%) | 18 (11.3%) | 36 (23.2%) |  |  |
| Mostly/Always - 4+5 | 419 (78.5%) | 86 (88.7%) | 82 (67.2%) | 139 (86.9%) | 112 (72.3%) |  |  |

## Supplementary material 4: HIRAID® Instructor survey results overall and by cluster

|  |  | **1** | **2** | **3** | **4** | **Total** |
| --- | --- | --- | --- | --- | --- | --- |
| **1. HIRAID Champions** | |  | **n(%)** | **n(%)** | **n(%)** | **n(%)** |
| **Were there HIRAID Champions at your site during the introduction of HIRAID?** | |  |  |  |  |  |
|  | (1) Yes | 23 (92%) | 24 (100%) | 11 (100%) | 30 (100%) | 88 (97.8%) |
|  | (0) No | 2 (8%) | 0 (0%) | 0 (0%) | 0 (0%) | 2 (2.2%) |
| **1. Track provision of HIRAID resources to staff** | |  |  |  |  |  |
|  | 4. Often/ Always |  | 16 (66.7%) | 2 (18.2%) | 18 (60%) | 36 (55.4%) |
|  | 3. Sometimes |  | 5 (20.8%) | 3 (27.3%) | 8 (26.7%) | 16 (24.6%) |
|  | 2. Rarely |  | 3 (12.5%) | 1 (9.1%) | 1 (3.3%) | 5 (7.7%) |
|  | 1. Never |  | 0 (0%) | 3 (27.3%) | 0 (0%) | 3 (4.6%) |
|  | 0. I am not sure |  | 0 (0%) | 2 (18.2%) | 3 (10%) | 5 (7.7%) |
| **Were HIRAID champions able to fulfill the tasks outlined ?** | |  |  |  |  |  |
|  | 4. Often/ Always | 10 (43.5%) | 10 (41.7%) | 3 (27.3%) | 20 (66.7%) | 43 (48.9%) |
|  | 3. Sometimes | 12 (52.2%) | 13 (54.2%) | 6 (54.5%) | 10 (33.3%) | 41 (46.6%) |
|  | 2. Rarely | 1 (4.3%) | 1 (4.2%) | 2 (18.2%) | 0 (0%) | 4 (4.5%) |
|  | 1. Never | 0 (0%) | 0 (0%) | 0 (0%) | 0 (0%) | 0 (0%) |
|  |  |  |  |  |  |  |
| **2. Track completion of face-to-face training** | |  |  |  |  |  |
|  | 4. Often/ Always |  | 15 (62.5%) | 5 (45.5%) | 19 (63.3%) | 39 (60%) |
|  | 3. Sometimes |  | 5 (20.8%) | 0 (0%) | 10 (33.3%) | 15 (23.1%) |
|  | 2. Rarely |  | 3 (12.5%) | 1 (9.1%) | 0 (0%) | 4 (6.2%) |
|  | 1. Never |  | 1 (4.2%) | 3 (27.3%) | 0 (0%) | 4 (6.2%) |
|  | 0. I am not sure |  | 0 (0%) | 2 (18.2%) | 1 (3.3%) | 3 (4.6%) |
| **Explain what HIRAID was to staff** | |  |  |  |  |  |
|  | 4. Often/ Always |  | 16 (66.7%) | 8 (72.7%) | 30 (100%) | 54 (83.1%) |
|  | 3. Sometimes |  | 8 (33.3%) | 2 (18.2%) | 0 (0%) | 10 (15.4%) |
|  | 2. Rarely |  | 0 (0%) | 1 (9.1%) | 0 (0%) | 1 (1.5%) |
|  | 1. Never |  | 0 (0%) | 0 (0%) | 0 (0%) | 0 (0%) |
|  | 0. I am not sure |  | 0 (0%) | 0 (0%) | 0 (0%) | 0 (0%) |
| **Answer questions staff had in relation to HIRAID** | |  |  |  |  |  |
|  | 4. Often/ Always |  | 18 (75%) | 6 (54.5%) | 23 (76.7%) | 47 (72.3%) |
|  | 3. Sometimes |  | 6 (25%) | 3 (27.3%) | 7 (23.3%) | 16 (24.6%) |
|  | 2. Rarely |  | 0 (0%) | 2 (18.2%) | 0 (0%) | 2 (3.1%) |
|  | 1. Never |  | 0 (0%) | 0 (0%) | 0 (0%) | 0 (0%) |
|  | 0. I am not sure |  | 0 (0%) | 0 (0%) | 0 (0%) | 0 (0%) |
| **Go through the documentation templates and requirements with staff** | |  |  |  |  |  |
|  | 4. Often/ Always |  | 13 (54.2%) | 7 (63.6%) | 19 (63.3%) | 39 (60%) |
|  | 3. Sometimes |  | 9 (37.5%) | 3 (27.3%) | 11 (36.7%) | 23 (35.4%) |
|  | 2. Rarely |  | 1 (4.2%) | 1 (9.1%) | 0 (0%) | 2 (3.1%) |
|  | 1. Never |  | 1 (4.2%) | 0 (0%) | 0 (0%) | 1 (1.5%) |
|  | 0. I am not sure |  | 0 (0%) | 0 (0%) | 0 (0%) | 0 (0%) |
| **Work with novice nurses to complete a patient assessment using HIRAID** | |  |  |  |  |  |
|  | 4. Often/ Always |  | 8 (33.3%) | 5 (45.5%) | 19 (63.3%) | 32 (49.2%) |
|  | 3. Sometimes |  | 9 (37.5%) | 5 (45.5%) | 9 (30%) | 23 (35.4%) |
|  | 2. Rarely |  | 7 (29.2%) | 1 (9.1%) | 1 (3.3%) | 9 (13.8%) |
|  | 1. Never |  | 0 (0%) | 0 (0%) | 1 (3.3%) | 1 (1.5%) |
|  | 0. I am not sure |  | 0 (0%) | 0 (0%) | 0 (0%) | 0 (0%) |
| **With novice nurses, practice handover and escalation using ISBAR and graded assertiveness** | |  |  |  |  |  |
|  | 4. Often/ Always |  | 6 (25%) | 4 (36.4%) | 11 (36.7%) | 21 (32.3%) |
|  | 3. Sometimes |  | 8 (33.3%) | 5 (45.5%) | 17 (56.7%) | 30 (46.2%) |
|  | 2. Rarely |  | 8 (33.3%) | 2 (18.2%) | 1 (3.3%) | 11 (16.9%) |
|  | 1. Never |  | 1 (4.2%) | 0 (0%) | 0 (0%) | 1 (1.5%) |
|  | 0. I am not sure |  | 1 (4.2%) | 0 (0%) | 1 (3.3%) | 2 (3.1%) |
| **Assist the nurse educator/clinical nurse educator to deliver the HIRAID education sessions to other staff** | |  |  |  |  |  |
|  | 4. Often/ Always |  | 7 (29.2%) | 0 (0%) | 16 (53.3%) | 23 (35.4%) |
|  | 3. Sometimes |  | 7 (29.2%) | 5 (45.5%) | 12 (40%) | 24 (36.9%) |
|  | 2. Rarely |  | 7 (29.2%) | 2 (18.2%) | 1 (3.3%) | 10 (15.4%) |
|  | 1. Never |  | 3 (12.5%) | 2 (18.2%) | 1 (3.3%) | 6 (9.2%) |
|  | 0. I am not sure |  | 0 (0%) | 2 (18.2%) | 0 (0%) | 2 (3.1%) |
| **How were HIRAID champions selected at your site?** | |  |  |  |  |  |
|  | (1) ALL volunteered | 12 (48%) | 13 (54.2%) | 2 (18.2%) | 6 (20%) | 33 (36.7%) |
|  | (2) SOME volunteered and some were asked | 8 (32%) | 10 (41.7%) | 6 (54.5%) | 24 (80%) | 48 (53.3%) |
|  | (3) NO ONE volunteered, all had to be asked | 5 (20%) | 1 (4.2%) | 3 (27.3%) | 0 (0%) | 9 (10%) |
| **I used the HIRAID documentation templates in my clinical documentation of assessment after triage** | |  |  |  |  |  |
|  | 4. Often/Always | 21 (84%) | 21 (87.5%) | 10 (90.9%) | 25 (83.3%) | 77 (85.6%) |
|  | 3. Sometimes | 2 (8%) | 2 (8.3%) | 0 (0%) | 5 (16.7%) | 9 (10%) |
|  | 2. Rarely | 2 (8%) | 0 (0%) | 1 (9.1%) | 0 (0%) | 3 (3.3%) |
|  | 1. Never | 0 (0%) | 1 (4.2%) | 0 (0%) | 0 (0%) | 1 (1.1%) |
| **2. HIRAID Education** | |  | **n(%)** | **n(%)** | **n(%)** |  |
| **Was the HIRAID PowerPoint* presentation used in educations sessions?** | |  |  |  |  |  |
|  | 4. Always | 12 (48%) | 13 (54.2%) | 11 (100%) | 24 (80%) | 60 (66.7%) |
|  | 3. Most of the time | 11 (44%) | 7 (29.2%) | 0 (0%) | 6 (20%) | 24 (26.7%) |
|  | 2. Hardly ever | 2 (8%) | 0 (0%) | 0 (0%) | 0 (0%) | 2 (2.2%) |
|  | 1. Never | 0 (0%) | 0 (0%) | 0 (0%) | 0 (0%) | 0 (0%) |
|  | 0. I don't know |  | 4 (16.7%) | 0 (0%) | 0 (0%) | 4 (4.4%) |
| **Was the education delivered by a person who completed a HIRAID train the trainer session (TtT)?** | |  |  |  |  |  |
|  | 4. Always | 23 (92%) | 23 (95.8%) | 11 (100%) | 28 (93.3%) | 85 (94.4%) |
|  | 3. Most of the time | 1 (4%) | 1 (4.2%) | 0 (0%) | 2 (6.7%) | 4 (4.4%) |
|  | 2. Hardly ever | 1 (4%) | 0 (0%) | 0 (0%) | 0 (0%) | 1 (1.1%) |
|  | 1. Never | 0 (0%) | 0 (0%) | 0 (0%) | 0 (0%) | 0 (0%) |
|  | 0. I don't know |  | 0 (0%) | 0 (0%) | 0 (0%) | 0 (0%) |
| **Did you make any changes to the Power point presentation that was used in the face to face education?** | |  |  |  |  |  |
|  | (1) presentation was delivered as provided | 18 (72%) | 24 (100%) | 11 (100%) | 26 (86.7%) | 79 (87.8%) |
|  | (2) 1-2 slides were removed | 4 (16%) | 0 (0%) | 0 (0%) | 3 (10%) | 7 (7.8%) |
|  | (3) > 2 slides were removed | 3 (12%) | 0 (0%) | 0 (0%) | 1 (3.3%) | 4 (4.4%) |
| **What parts of the presentation were changed/ removed.** | |  |  |  |  |  |
|  | 10. Change in content i.e removed and/ or added further details | 7 (28%) | 0 (0%) | 0 (0%) | 3 (10%) | 10 (11.1%) |
|  | 11. Change in order/ flow of slides | 0 (0%) | 0 (0%) | 0 (0%) | 2 (6.7%) | 2 (3.1%) |
|  | 9. other | 0 (0%) | 0 (0%) | 0 (0%) | 0 (0%) | 0 (0%) |
| **3. HIRAID Communication** | |  | **n(%)** | **n(%)** | **n(%)** |  |
| **Nursing staff received communication from the ED NUM or educator reinforcing the use of HIRAID in the ED.** | |  |  |  |  |  |
|  | 4. Often/ Always | 18 (72%) | 18 (75%) | 7 (63.6%) | 21 (70%) | 64 (71.1%) |
|  | 3. Sometimes | 4 (16%) | 6 (25%) | 4 (36.4%) | 9 (30%) | 23 (25.6%) |
|  | 2. Rarely | 2 (8%) | 0 (0%) | 0 (0%) | 0 (0%) | 2 (2.2%) |
|  | 1. Never | 1 (4%) | 0 (0%) | 0 (0%) | 0 (0%) | 1 (1.1%) |
| **How was the communication reinforcing HIRAID use delivered?** | |  |  |  |  |  |
|  | 1. Email |  | 18 (75%) | 8 (72.7%) | 26 (86.7%) | 52 (80%) |
|  | 2. Staff Huddle |  | 15 (62.5%) | 7 (63.6%) | 28 (93.3%) | 50 (76.9%) |
|  | 3. Meetings |  | 8 (33.3%) | 3 (27.3%) | 7 (23.3%) | 18 (27.7%) |
|  | 4. Face-to-face |  | 21 (87.5%) | 10 (90.9%) | 19 (63.3%) | 50 (76.9%) |
|  | 5. Newsletter |  | 1 (4.2%) | 1 (9.1%) | 3 (10%) | 5 (7.7%) |
|  | 6. Other |  | 0 (0%) | 1 (9.1%) | 1 (3.3%) | 2 (3.1%) |
| **Were the results of these audits communicated to ED nursing staff?** | |  |  |  |  |  |
|  | 4. Often/ Always | 13 (52%) | 17 (70.8%) | 6 (54.5%) | 20 (66.7%) | 56 (62.2%) |
|  | 3. Sometimes | 7 (28%) | 6 (25%) | 4 (36.4%) | 9 (30%) | 26 (28.9%) |
|  | 2. Rarely | 3 (12%) | 1 (4.2%) | 0 (0%) | 0 (0%) | 4 (4.4%) |
|  | 1. Never | 2 (8%) | 0 (0%) | 1 (9.1%) | 1 (3.3%) | 4 (4.4%) |
| **HIRAID posters were displayed in the ED** | |  |  |  |  |  |
|  | (0) no posters | 11 (44%) | 9 (37.5%) | 3 (27.3%) | 6 (20%) | 29 (32.2%) |
|  | (1) 1-2 posters | 10 (40%) | 10 (41.7%) | 1 (9.1%) | 9 (30%) | 30 (33.3%) |
|  | (2) 3-4 posters | 3 (12%) | 3 (12.5%) | 5 (45.5%) | 8 (26.7%) | 19 (21.1%) |
|  | (3) > 4 posters | 1 (4%) | 2 (8.3%) | 2 (18.2%) | 7 (23.3%) | 12 (13.3%) |
| **4. HIRAID Feedback** | |  | **n(%)** | **n(%)** | **n(%)** |  |
| **Did you access any of these supports provided in the implementation of HIRAID?** | |  |  |  |  |  |
|  | 1. HIRAID CNC |  | 19 (73.1%) | 11 (91.7%) | 28 (93.3%) | 58 (89.2%) |
|  | 2. Microsoft Teams chat |  | 12 (46.2%) | 4 (33.3%) | 2 (6.7%) | 18 (27.7%) |
|  | 3. Virtual drop in sessions |  | 5 (19.2%) | 2 (16.7%) | 3 (10%) | 10 (15.4%) |
|  | 4. Other support |  | 2 (7.7%) | 0 (0%) | 1 (3.3%) | 3 (4.6%) |

## Supplementary material 5: Heat Map of implementation spread and scale


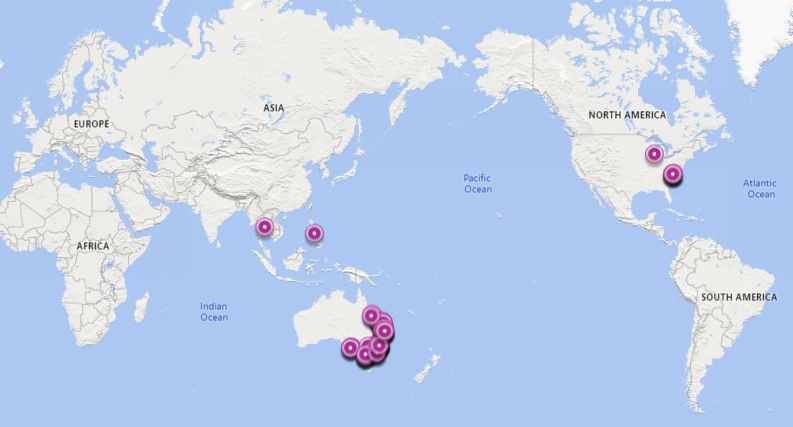

Supplement: Supplementary file 1 — Supplementary Material 1. [file 13012_2024_1383_MOESM1_ESM.docx]
